# Study on Photocatalytic Degradation of NO Gas by Cement Paste Incorporated with Nano-TiO_2_

**DOI:** 10.3390/ma19153214

**Published:** 2026-07-28

**Authors:** Zigeng Wang, Tong Liu, Yue Li, Chenwei Gu

**Affiliations:** 1 Key Laboratory of Urban Security and Disaster Engineering of Ministry of Education, Beijing Key Laboratory of Earthquake Engineering and Structural Retrofit, College of Architecture and Civil Engineering, Beijing University of Technology, Beijing 100124, China; zigengw@bjut.edu.cn (Z.W.); ltong@emails.bjut.edu.cn (T.L.); 2 State Key Laboratory of Mountain Bridge and Tunnel Engineering, Chongqing Jiaotong University, Chongqing 400074, China; 3 China Road & Bridge Corporation, Beijing 100011, China; guchenwei@crbc.com

**Keywords:** cement, Nano-TiO_2_, photocatalytic degradation, NO gas, microstructural analysis

## Abstract

Nitrogen oxides (NOx) emitted from anthropogenic sources pose severe threats to urban environments. While incorporating nano-TiO_2_ into cementitious materials offers a promising photocatalytic remediation strategy, the coupled effects of pore-structure modification and curing age on the degradation efficiency remain underexplored. This study systematically investigated the NO gas degradation performance of nano-TiO_2_-modified cement paste by controlling the TiO_2_ content (3–12%), air-entraining agent content, water-cement ratio, and curing age. The results indicate that an optimal nano-TiO_2_ content of 6% yields the highest compressive strength. Meanwhile, higher nano-TiO_2_ content (up to 12%) significantly enhances NO removal (reaching 9.73%), but excessive air-entraining agents trap the photocatalyst, thereby reducing efficiency. Furthermore, increasing the water-cement ratio promotes gas diffusion, whereas prolonged curing densifies the matrix and obstructs UV-catalyst contact, decreasing the removal ratio by 20.3% at 28 days. Microstructural analyses elucidate that NO is photocatalytically oxidized into nitrate ions (NO_3_^−^) without degrading the essential C–S–H gel. This study provides critical insights into the microstructural optimization of photocatalytic cements, offering a practical design framework for photocatalytic cements with potential for long-term environmental application, pending durability validation.

## 1. Introduction

With the rapid development of modern society, nitrogen oxides (NOx, primarily NO and NO_2_), originating from both anthropogenic sources (such as vehicle exhaust combustion and industrial facility emissions) and natural sources (e.g., lightning, soil microbial processes, wildfires), have led to increasingly severe climate and environmental issues.

The photocatalytic materials were “green catalysts” that harnessed light energy to drive chemical reactions. Their core mechanism involved absorbing light energy to generate highly reactive electrons and holes, thereby initiating redox reactions. There were numerous photocatalytic materials, such as TiO_2_, CdS, Ag_3_PO_4_, ZnO, ZrO_2_, CeO_2_, and others [1]. The two most commonly used materials were nano-ZnO and nano-TiO_2_. Among these, nano-ZnO offers numerous advantages such as high electron mobility, excellent light stability, non-toxicity, and good chemical stability. Although ZnO and TiO_2_ shared the same bandgap (Eg = 3.2 eV) [2]. However, ZnO exhibited a certain degree of retarding effect, and its photocatalytic activity was significantly lower than that of nano-TiO_2_, thus severely limiting its application in cement-based materials. Due to the exceptionally high efficiency in treating nitrogen oxides (NOx) gases, TiO_2_ was commonly used as a photocatalyst [3,4,5]. When nano-TiO_2_ was exposed to ultraviolet light, it could generate various free radicals with potent redox capabilities, such as hydroxyl radicals (-OH) and superoxide anions (O^2−^). These free radicals rapidly reacted with airborne pollutants, breaking them down into harmless substances to achieve air purification [3]. Moreover, TiO_2_’s photochemical stability, chemical inertness without ultraviolet (UV) irradiation, non-toxicity, safety, and stability in cement-based materials made it a suitable photocatalyst [5,6,7].

The cementitious materials held significant importance in civil engineering due to their extensive application in construction materials, and their strong bonding capacity, excellent workability, and outstanding durability made them an ideal substrate for loading photocatalysts [8]. The cementitious materials are typically porous and heterogeneous, and the nano-TiO_2_ can improve the microstructure of cementitious materials at the nanoscale, thereby enhancing their macroscopic properties [9,10,11,12]. Some literature reports indicated that the nanomaterials can enhance the mechanical strength, durability, and ductility of cementitious materials such as cement and can accelerate the hydration reaction [13,14]. Moreover, incorporating photocatalysts into the cementitious materials can effectively enhance their photocatalytic activity. Similarly, the porous characteristics of the cementitious materials provided numerous fixation sites for photocatalysts, and their extensive exposed surface area has been reported as a target carrier for these photocatalysts [15,16,17,18,19,20]. Since the first report on the photocatalytic activity of nano-TiO_2_ in water-splitting reactions, extensive studies have been conducted on the photocatalytic degradation of environmental pollutants using nano-TiO_2_. In particular, nano-TiO_2_ has been widely applied in cementitious materials to develop functional building materials with pollutant removal capability [21,22]. Based on the aforementioned principles, researchers combined TiO_2_ with cementitious materials to create a novel nano-TiO_2_-reinforced cementitious matrix. This material retained the inherent properties of the cementitious materials while gaining the ability to efficiently degrade air pollutants due to the incorporation of nano-TiO_2_. The nano-TiO_2_-reinforced cementitious matrix has been proven effective in purifying the environment by degrading nitrogen oxides (NOx) [23,24,25]. These photocatalytic cements have been tested and demonstrated atmospheric purification activity and self-cleaning capabilities. In practical applications, they offer new approaches for improving urban air quality. For instance, in road paving and exterior wall construction, they demonstrated notable air purification effects [3,26].

Numerous studies [27,28,29,30,31] have conducted in-depth investigations into the photocatalytic performance, self-cleaning properties, mechanical properties, and durability of the cementitious materials modified with nano-TiO_2_ powders. For instance, Zhen et al. conducted more in-depth research on the effect of different TiO_2_ nano-doping levels on the compressive strength of cement-based materials. The results indicated that a 4% TiO_2_ content significantly enhanced the compressive strength of cement-based materials [32]. Li et al. investigated the effect of TiO_2_ particle size on the cementitious material properties, finding that smaller TiO_2_ particles (10 nm) exhibited a more pronounced enhancement effect on the mechanical properties and hydration nucleation, compared to larger particles (15 mm) [33]. These studies not only confirmed the feasibility of cement reinforced with nano-TiO_2_, but also provided valuable insights for subsequent research.

In recent years, significant progress has been achieved in the development of photocatalytic cementitious materials for atmospheric pollutant removal. Recent studies have demonstrated that the incorporation of nano-TiO_2_ can effectively improve the degradation efficiency of nitrogen oxides (NOx), volatile organic compounds (VOCs), and other airborne contaminants [34,35,36]. Researchers have focused not only on enhancing photocatalytic activity through optimized TiO_2_ dosage and particle dispersion, but also on improving the long-term durability and environmental adaptability of photocatalytic cement-based materials [37,38]. Recent reviews have highlighted that factors such as ultraviolet irradiation intensity, environmental humidity, pollutant concentration, and surface exposure conditions significantly influenced photocatalytic performance. Furthermore, several field-scale investigations have confirmed the practical potential of photocatalytic concrete pavements and façade materials for urban air purification applications, demonstrating increasing interest in translating laboratory-scale findings into real engineering practice [36,38].

Recent work has expanded the use of photocatalytic cementitious materials from NOx control to the removal of volatile organic compounds and other airborne pollutants [34,35,36]. Current optimization strategies focus on TiO_2_ dosage and dispersion while also considering service durability and environmental adaptability [37,38]. The incorporation method provided improved structural compatibility and better resistance against catalyst loss during long-term service. However, the photocatalytic efficiency of incorporated nano-TiO_2_ was often restricted because a considerable proportion of TiO_2_ particles are embedded within hydration products, resulting in limited exposure to ultraviolet light and gaseous NOx molecules [39].

More recently, attention has shifted toward understanding the influence of microstructural characteristics on photocatalytic efficiency. Pollutant adsorption, intrapaste diffusion, and contact with photoactive sites are strongly influenced by total porosity, pore-size distribution, and pore connectivity [40,41,42]. Parameters such as porosity, pore connectivity, and pore size distribution can substantially affect the transport of gaseous pollutants within the cement matrix and consequently influence the overall photocatalytic degradation efficiency [40,41]. Recent studies have further highlighted long-term performance, environmental safety, and multifunctional applications of photocatalytic cementitious materials [43,44]. Recent reviews published since 2023 have further demonstrated that nano-TiO_2_ can simultaneously influence cement hydration, microstructural evolution, mechanical performance, durability, and photocatalytic functionality. These reviews also identified preparation methods, photocatalyst dosage, mechanical compatibility, and life-cycle performance as key design considerations for photocatalytic cementitious materials [45].

In addition to laboratory-scale optimization, increasing attention has been paid to the long-term performance, environmental sustainability, and practical application of photocatalytic cementitious materials, particularly TiO_2_-modified systems for atmospheric pollutant removal [3,6,8]. The photocatalytic efficiency of these materials is governed not only by the intrinsic activity of TiO_2_, but also by the exposure of TiO_2_ particles within the cement matrix, the characteristics of the pore structure, and gas-phase mass transport [3,9]. The composition and particle-size distribution of nano-TiO_2_ dispersions can further affect the accessibility of photocatalytically active sites. Highly dispersed nano-TiO_2_ particles may promote cement hydration by acting as nucleation sites. However, the subsequently formed hydration products can partially encapsulate the TiO_2_ particles, thereby limiting their exposure to ultraviolet irradiation and gaseous pollutants [36]. Consistent with this mechanism, Jia et al. [3] demonstrated that the adsorption and reaction kinetics of NO were strongly influenced by mass-transfer resistance within the pore network. Therefore, pore-structure parameters, including porosity, pore connectivity, and pore-size distribution, are key factors controlling pollutant diffusion and photocatalytic performance in cementitious systems [9].

Long-term exposure studies have further indicated that photocatalytic activity may decrease with service age due to progressive hydration, carbonation, and surface coverage of TiO_2_ particles by hydration products [9,11]. Wang et al. [11] reported that microstructural densification during cement hydration improved mechanical properties but may simultaneously restrict transport pathways and reduce active surface accessibility, thereby limiting photocatalytic efficiency in aged materials.

Accordingly, pore structure regulation has been recognized as an effective approach to enhance photocatalytic performance by improving gas diffusion and increasing the probability of contact between pollutants and active TiO_2_ sites [3]. Hüsken et al. [6] and subsequent studies have shown that increasing accessible porosity can significantly enhance NOx degradation efficiency in photocatalytic concrete. However, excessive porosity may also compromise mechanical integrity and durability, indicating a necessary balance between reactivity and structural performance [11]. Porous recycled aggregates have also been investigated as carriers for nano-TiO_2_ because their rough and porous surfaces can facilitate photocatalyst loading and increase the accessibility of active sites [46]. However, such surface-treated aggregate systems differ from the bulk-incorporated cement paste investigated in the present study. Recent quantitative analyses further indicated that NO removal was closely related to TiO_2_ coverage on the photoactive surface, whereas pore-structure characteristics can influence NO_2_ formation and reaction selectivity. This suggested that pore optimization should account for both pollutant transport and product selectivity rather than removal efficiency alone [47].

In terms of environmental safety, several studies have confirmed that nano-TiO_2_ particles were strongly immobilized within calcium silicate hydrate (C–S–H) gels formed during cement hydration, which significantly limited nanoparticle release during service life [48]. This encapsulation effect has been identified as a key mechanism ensuring the environmental compatibility of nano-TiO_2_-modified cementitious materials under realistic exposure conditions [49].

Furthermore, life-cycle and sustainability assessments have suggested that photocatalytic cement-based materials can contribute to urban air quality improvement through continuous NOx removal and may partially offset their environmental footprint under long-term service conditions [10]. Recent low-carbon binder studies have also shown that the environmental value of photocatalytic composites depended on the combined assessment of binder carbon footprint, abrasion resistance, and NOx-purification performance [50]. Such benefits were particularly relevant for large-scale applications such as pavements and building facades exposed to solar irradiation.

Recent studies have also extended photocatalytic cementitious systems from single NOx degradation toward broader multi-pollutant applications, including volatile organic compounds (VOCs) and other airborne contaminants [7]. In parallel, the development of visible-light-responsive photocatalysts and composite semiconductor systems has been proposed to overcome the limitation of UV-only activation, thereby improving applicability under natural solar conditions [8]. These advances highlighted the importance of understanding the coupled interactions among pore structure evolution, photocatalyst accessibility, and pollutant transport behavior in cementitious matrices. Recent reviews further identified particle agglomeration, aging-induced activity loss, and insufficient photocatalyst–matrix interfacial bonding as major obstacles to practical application, while ultrasonic dispersion, composite modification, and functional surface treatments were regarded as promising optimization strategies [51].

Field-scale studies have emphasized that laboratory activity does not necessarily translate directly to outdoor performance. A recent wall-coating investigation under real-life conditions observed measurable NOx abatement, but accelerated weathering and immersion–drying cycles caused substantial activity losses, highlighting the need to evaluate durability under realistic exposure conditions. More recently, TiO_2_-coated permeable concrete combining high interconnected porosity with durable surface fixation retained most of its initial activity after accelerated weathering and demonstrated NOx removal under natural outdoor illumination, illustrating the potential of pore-rich substrates for practical urban applications [52].

Recent material-level studies further showed that the performance of nano-TiO_2_-containing cementitious systems depended on the physicochemical state of the particles and their interaction with the host matrix. XRD/Rietveld and microstructural analysis of nano-TiO_2_-modified Portland cement mortar has demonstrated that nano-TiO_2_ addition can influence phase assemblage and microstructural characteristics [53]. At the dispersion stage, the applied protocol can alter the physicochemical identity and toxicity profile of TiO_2_ suspensions [54], while the colloidal size distribution has been shown to affect photocatalytic degradation behavior [55]. Evidence from related nano-titanium-enriched glass-ionomer cement systems also indicated that titanium-containing nanoparticles can modify mechanical and interfacial properties [56]. Together, these findings underline that TiO_2_ dosage alone is insufficient to describe material performance. The particle dispersion, size distribution, and matrix interaction must also be considered.

Recent publications have further clarified why the photocatalytic performance of nano-TiO_2_ cement-based materials cannot be attributed solely to TiO_2_ dosage or intrinsic activity. Reviews of adsorption kinetics and recent advances indicated that NOx removal was governed by the combined processes of pollutant adsorption, surface reaction, and mass transfer through the cementitious matrix [3,57]. At the same time, experimental evidence showed that nano-TiO_2_ modified cement hydration, microstructural development, and mechanical performance, thereby changing the exposure and accessibility of photocatalytically active sites during curing [58]. Beyond photocatalytic efficiency, the practical use of these materials also required attention to the ecotoxicity of leachates and the potential release of nanoparticles under abrasion [59,60]. The importance of active-site accessibility was further demonstrated by comparisons between intermixed and spray-coated TiO_2_ concrete, in which differences in catalyst exposure led to distinct NOx degradation performance [61]. Consistent with these findings, a recent comprehensive review emphasized that particle dispersion, hydration evolution, photocatalyst exposure, mechanical compatibility, durability, and environmental safety should be evaluated together [62].

At the molecular and phase levels, FTIR studies of early Portland cement hydration [63], water under adsorption and confinement conditions [64], and fresh C–S–H gels [65], together with observations of phase transformations during cement hydration [66], provided the analytical basis for identifying hydration-related changes. These studies are relevant because hydration-product evolution can alter matrix densification, pore-related transport pathways, and the extent to which TiO_2_ surfaces remain accessible to irradiation and gaseous NO.

Despite these advances, the factors controlling photocatalytic performance have generally been examined separately and under different cementitious matrices, incorporation methods, curing regimes, and test conditions. Consequently, the relative roles of TiO_2_ dosage, gas-transport-related mixture parameters, hydration-induced matrix evolution, and active-site accessibility remain difficult to distinguish. In particular, limited information is available on how air-entraining-agent dosage, water-to-cement ratio, and curing age affect NO removal within the same bulk-incorporated nano-TiO_2_ cement-paste system. These factors may exert competing effects: improved gas-transport pathways can facilitate NO diffusion toward exposed TiO_2_ surfaces, whereas continued hydration and matrix densification may restrict pollutant transport and partially cover active sites. The resulting balance between photocatalytic efficiency and mechanical performance therefore remains insufficiently clarified.

During curing, ongoing hydration reactions continuously modified the microstructure and may further affect the exposure state of nano-TiO_2_ particles by forming additional hydration products that partially cover active surfaces [9]. As a result, the coupled effects of pore structure evolution, curing age, and pollutant degradation behavior remain insufficiently understood. Therefore, further investigation into the relationship between pore structure characteristics and photocatalytic NO degradation behavior is essential for optimizing the design of durable and efficient photocatalytic cementitious materials.

Although research on the photocatalytic performance of cement paste doped with nano-TiO_2_ has achieved certain results, further investigation is needed into how the pore structure of the cement affects the photocatalytic performance of the nano-TiO_2_. Therefore, this study employed XRD, FTIR spectroscopy, and TG-DSC tests to apply nano-TiO_2_-modified cement paste in photocatalysis. By altering the water-cement ratio and incorporating air-entraining agents to modify the pore structure of the cement paste, the mechanical and photocatalytic degradation performance for NO gas were investigated. Unlike previous studies that generally examined TiO_2_ dosage, pore characteristics, or curing conditions separately and frequently used different cementitious matrices and testing conditions, the present study compared these influencing factors within the same bulk-incorporated nano-TiO_2_ cement-paste system under consistent preparation and photocatalytic testing conditions. The new scientific knowledge provided by this work lay not merely in identifying an optimum nano-TiO_2_ dosage, but in clarifying how photocatalyst dosage, pore-related gas-transport conditions, hydration-induced matrix densification, and active-site accessibility jointly govern NO-removal behavior. The results further revealed a curing-age-dependent trade-off in which progressive hydration improved matrix compactness and mechanical performance but simultaneously restricted gaseous pollutant transport and partially reduced the accessibility of TiO_2_ active sites, thereby providing a mechanistic basis for balancing mechanical and photocatalytic performance in TiO_2_-modified cementitious materials.

## 2. Materials and Methods

### 2.1. Materials and Mix Proportions

#### 2.1.1. Raw Materials

**Figure 1:**
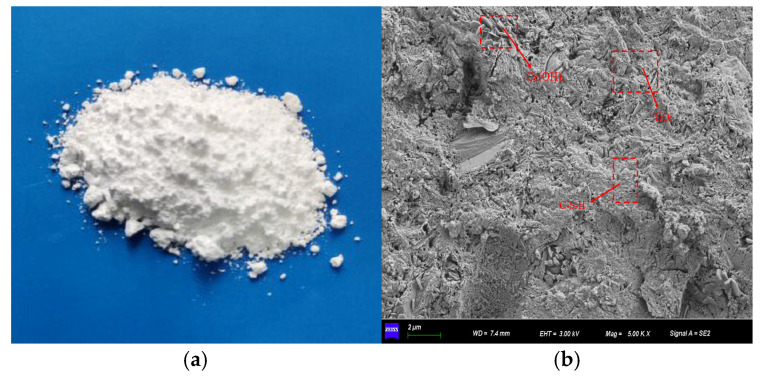

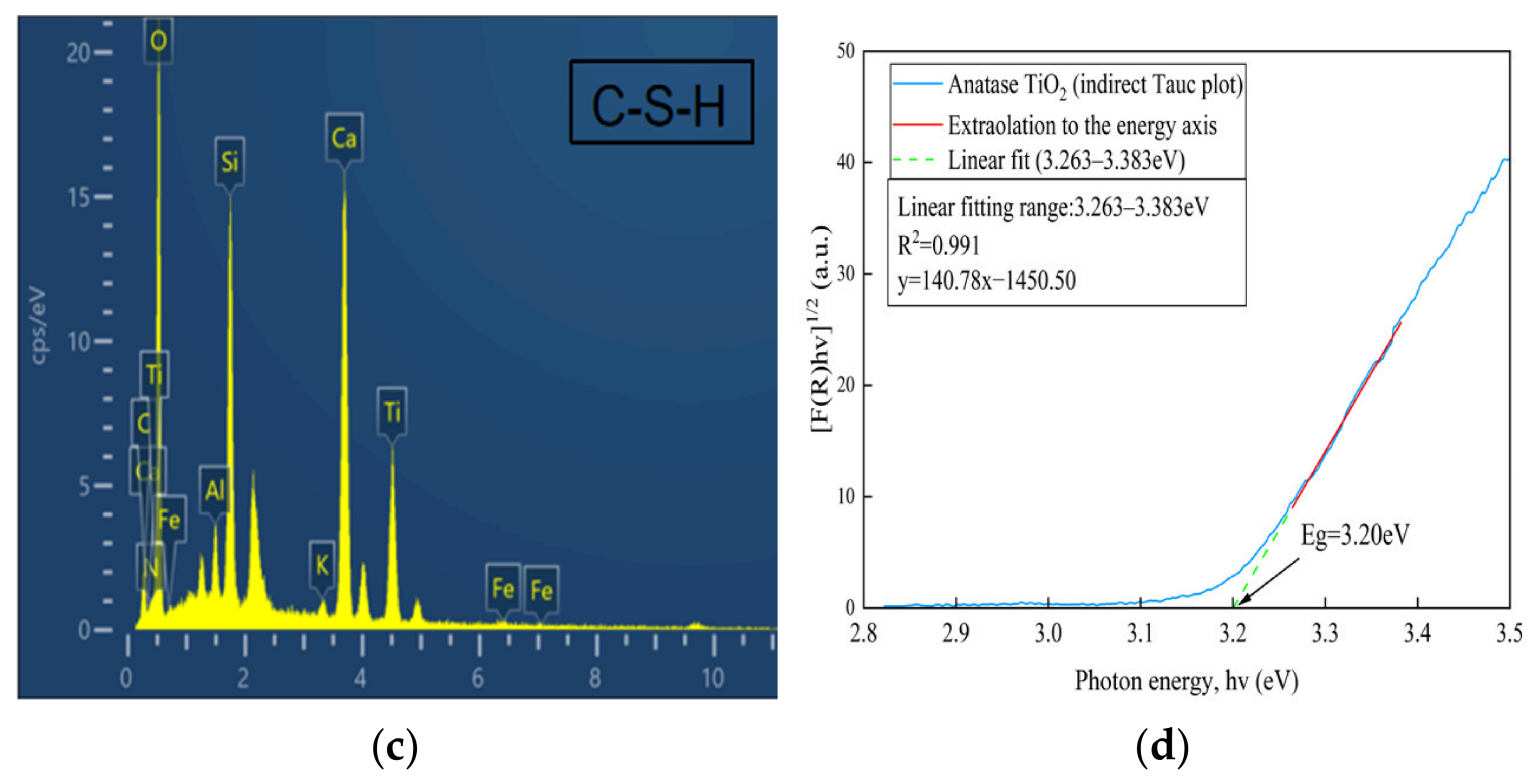
Morphological, elemental, and optical characterization of nano-TiO_2_ and nano-TiO_2_-modified cement composite: (**a**) morphology of pristine nano-TiO_2_ powder; (**b**) SEM image of the hydrated nano-TiO_2_-modified cement composite; (**c**) EDS elemental mapping of the nano-TiO_2_-modified cement composite; and (**d**) Indirect Tauc plot of anatase nano-TiO_2_. The absorption-edge region from 3.263 to 3.383 eV was used for linear regression, and extrapolation of the fitted line to the photon-energy axis yielded Eg = 3.20 eV (R^2^ = 0.991).

The experimental program used ordinary Portland cement, nano-TiO_2_, a polycarboxylate water reducer, an air-entraining agent, and deionized water. The cement was a 42.5 R product supplied by Anhui Conch Cement Co., Ltd., Wuhu city, China; its chemical composition, mineral phases, and physical properties are listed in Table 1, Table 2 and Table 3. The commercial nano-TiO_2_ was obtained from Ningbo Jiemina New Materials Technology Co., Ltd., Ningbo city, China and consisted mainly of anatase, with a primary crystallite size of about 12 nm. The anatase phase and crystallite size were verified by XRD, and the latter was calculated using the Scherrer equation [53]. The optical band gap of the commercial nano-TiO_2_ powder was experimentally determined using UV–Vis diffuse reflectance spectroscopy (UV–Vis DRS; UV-2600, Shimadzu, Kyoto city, Japan) over a wavelength range of 200–800 nm, with BaSO_4_ used as the reference material. The measured diffuse reflectance data were converted using the Kubelka–Munk function, F(R) = (1 − R)^2^/(2R), where R is the diffuse reflectance. Considering the indirect allowed transition of anatase TiO_2_, [F(R)hv]^1/2^ was plotted against the photon energy hν. As shown in Figure 1d, the approximately linear absorption-edge region from 3.263 to 3.383 eV was fitted using least-squares linear regression. The regression yielded y = 140.78hν − 450.50, with a coefficient of determination of R^2^ = 0.991. Extrapolation of the fitted line to [F(R)hν]^1/2^ = 0 gave an indirect optical band gap of approximately 3.20 eV. The photon energy of the incident UV light was calculated according to E = 1240/λ, where E is the photon energy (eV) and λ is the wavelength (nm). For the 365 nm irradiation source, the calculated photon energy was approximately 3.40 eV, which was higher than the band gap energy of anatase nano-TiO_2_ (~3.2 eV), indicating that the applied UV irradiation was sufficient to excite electron-hole pairs in nano-TiO_2_ and initiate photocatalytic redox reactions.

**Figure 2:**
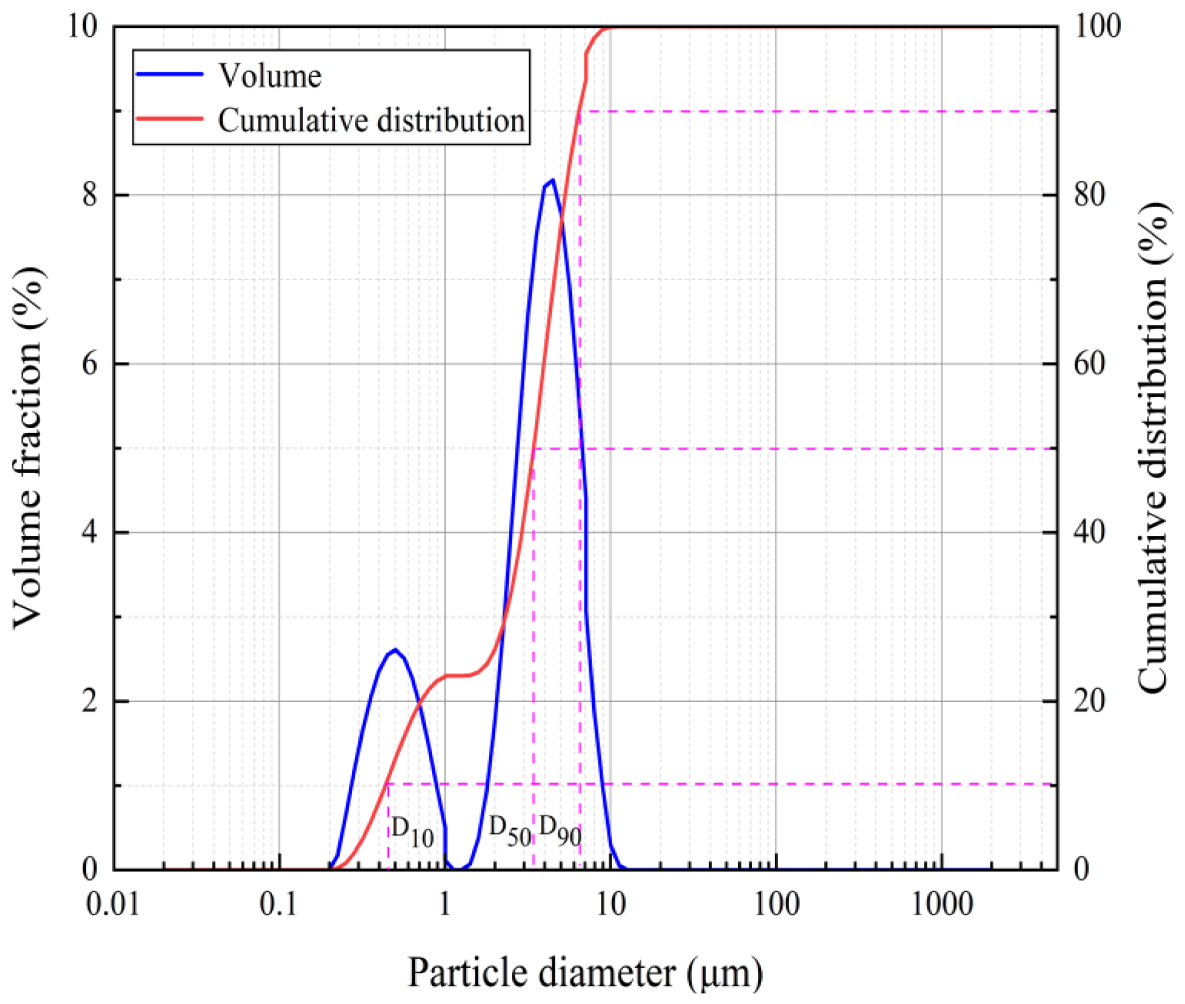
The particle size distribution of nano-TiO_2_.

As shown in Figure 1a, the pristine nano-TiO_2_ particles exhibited nanoscale morphology. Figure 1b presents the SEM image of the nano-TiO_2_-modified cement composite after hydration, rather than pure nano-TiO_2_ particles. The corresponding EDS elemental mapping (Figure 1c) confirmed the coexistence of Ti, O, Ca, and Si elements, indicating the incorporation of nano-TiO_2_ into the cement hydration matrix. Laser-diffraction measurements gave D10, D50, and D90 values of 0.489, 4.310, and 8.089 μm, respectively (Figure 2). These micrometer-scale distribution values are larger than the primary crystallite size because high-surface-energy nanoparticles form agglomerates in dry powder and aqueous suspension. In this paper, “nano-TiO_2_” therefore refers to the primary crystalline particles, although they may exist as larger agglomerates in the cement paste. The polycarboxylate water reducer was supplied by Jiangsu Subo New Material Co., Ltd., Nanjing city, China and had a solid content of 30%, a density of 1.06 g/mL, a pH of 8.9, and a stated water-reduction rate of 35%. The AE-11 liquid air-entraining agent, supplied by Shanghai Yingshan New Material Technology Co., Ltd., Shanghai city, China had a density of 0.97 g/L, an effective content of 28%, and an air content of 5.5%. This air-entraining agent was an anionic surfactant that significantly reduced water surface tension and interfacial energy, exhibiting excellent air-entraining and bubble-stabilizing effects. The water used for the tests was deionized water.

#### 2.1.2. Mix Proportions

Eleven mixtures were prepared to separate the effects of nano-TiO_2_ dosage, air-entraining agent dosage, and water-cement ratio on NO removal (Table 4). The water reducer was fixed at 2% of the cement mass. Mixtures C0-0-0.4, C3-0-0.4, C6-0-0.4, C9-0-0.4, and C12-0-0.4 contained 0%, 3%, 6%, 9%, and 12% nano-TiO_2_, respectively. As discussed in Section 3.2, the 28 d compressive strength decreased when the nano-TiO_2_ dosage exceeded 6%. Therefore, 6% nano-TiO_2_ was selected for the series used to study the other mixture variables. The air-entraining-agent series contained 0%, 0.05%, 0.10%, 0.15%, and 0.20% admixture and was designated C6-0-0.4 to C6-0.2-0.4. The water-cement-ratio series comprised C6-0-0.35, C6-0-0.4, and C6-0-0.45, corresponding to ratios of 0.35, 0.40, and 0.45.

### 2.2. Sample Preparation and Curing

Nano-TiO_2_ was first dispersed ultrasonically to reduce agglomeration before it was mixed with cement. The required amounts of nano-TiO_2_, water, and water reducer were combined to form a suspension and treated in an ultrasonic cell disruptor for 10, 20, or 30 min. Each pulse cycle consisted of 6 s of sonication followed by a 2 s pause. After treatment, liquid was sampled from the center of the suspension and analyzed spectrophotometrically. The dispersion time selected from these measurements is discussed in Section 3.1.

After ultrasonic treatment, the prescribed amount of air-entraining agent was added to the suspension and mixed manually for 60 s. Cement and the prepared suspension were then placed in the mixing bowl. Mixing consisted of 60 s at 60 rpm, a 30 s rest, and 90 s at 120 rpm. The fresh paste was poured into six-cavity steel molds measuring 40 mm × 40 mm × 40 mm. Each mold was sealed with plastic film and stored in a standard curing chamber at 20 ± 2 °C and relative humidity above 95% until the specified age. The specimens were subsequently used for compressive-strength and photocatalytic tests.

For phase and thermal analyses, 28 d specimens were fractured after mechanical testing, and pieces taken at least 5 mm from the exposed surface were immersed in anhydrous ethanol for 48 h to stop hydration. The pieces were then vacuum-dried at 40 °C and −0.1 MPa until their mass became constant. After 30 min of planetary ball milling, the powder was passed through a 0.056 mm sieve and used for XRD, FTIR, and TG-DSC measurements.

### 2.3. Macro Performance Test

#### 2.3.1. Compressive Strength

Compressive strength was determined at 28 d in accordance with GB/T 17671-1999 [67], which follows the ISO method for cement strength testing. Six specimens from each of the five nano-TiO_2_ dosage groups were loaded with a DYE-300S automatic compression/flexure machine, Beijing Zhongtong Jianyi Instrument equipment Co. LTD, Beijing city, China and the mean value was reported. The machine capacity was 300 kN, and the loading rate was 2.4 kN/s.

#### 2.3.2. Photocatalytic Degradation of NO Gas

The photocatalytic degradation performance of NO gas in 11 groups of cement paste samples was tested using a 42i-TL trace nitrogen oxide analyzer manufactured by Thermo Fisher Scientific Inc., Waltham city, MA, USA. The instrument was equipped with an 8 W UV lamp, a photocatalytic polymer glass reactor measuring 30 cm × 15 cm × 10 cm, and a humidification chamber. The NO gas concentration measurement range was 0–1000 ppb. The 8 W UV lamp utilized in the reactor emitted a dominant wavelength of 365 nm. The irradiance at the sample surface was measured at 1.5 W/m^2^. The zero-point noise was 0.20 ppb rms. The standard response time was 5 s. While natural sunlight contains a broader spectrum with a lower proportional UV intensity (typically 3–5%), this controlled UV setup ensures accelerated and standardized testing of the intrinsic photocatalytic capacity. The ambient temperature and relative humidity inside the reactor were strictly controlled at 25 ± 2 °C and 50 ± 5%, respectively, ensuring high reproducibility of the testing environment. The specific test procedure was as follows: Three cubic samples per group were placed in the transparent reaction chamber of the NOx analyzer. For each mixture, the complete photocatalytic test was independently repeated three times under identical operating conditions. In each independent test, three cubic samples from the same mixture were placed simultaneously in the reactor. Before each test, the reactor was restored to the same initial conditions, and adsorption–desorption equilibrium was re-established before UV irradiation. This chamber, constructed from polymer glass and covered with acrylic, formed a rectangular reactor with a volume of 4.5 L. The 8 W UV lamp was positioned directly above the reactor. The NO gas was then slowly introduced. The gas stream was fully premixed by a gas mixer, with flow rates for the gas stream and NO controlled by mass flow controllers at 2.4 L/min and 24 mL/min, respectively. The NO gas was diluted to approximately 800 ppb by the gas stream. The relative humidity of the NO gas was controlled at 50% by passing the gas stream through the humidification chamber first. After reaching adsorption–desorption equilibrium, the UV lamp was turned on for a 30 min reaction. The concentration change in the NO gas was continuously measured using a NOx analyzer, Beijing Zhongke Boda Instrument Technology Co. LTD, Beijing city, China to determine the photocatalytic degradation capacity of the sample under UV light.

### 2.4. Microscopic Test

#### 2.4.1. Ultrasonic Dispersion of Nano-TiO_2_ Suspension

The nano-TiO_2_ suspension underwent ultrasonic dispersion using the JY96-IIN ultrasonic cell disruptor from Hangzhou Jingfei Instrument Technology Co., Ltd., Hangzhou city, China. The device operated at a frequency range of 20–25 kHz, equipped with a random amplitude rod Φ6. The ultrasonic power ranged from 1.5 to 150 W, with single pulse duration and interval time continuously adjustable between 0.1 and 99.9 s. Dispersion was evaluated with a 752 G UV-Vis spectrophotometer (Shanghai Yidian Analytical Instrument Co., Ltd., Shanghai city, China) over 200–1000 nm, with a wavelength accuracy of ±2 nm.

#### 2.4.2. XRD

X-ray diffraction was conducted to determine the phase assemblages of the specimens. The measurements were performed on an X’Pert PRO MPD diffractometer, Malvern Panalytical, Almelo cigy, Netherland, operating with Cu Kα radiation at an accelerating voltage of 40 kV. Diffraction patterns were collected within a 2θ range of 10–80°, using an angular increment of 0.02° and a scanning rate of 2°/min. After measurement, the recorded patterns were imported into Jade 6.5, where the characteristic peaks were compared with standard diffraction data to assign the corresponding crystalline phases.

#### 2.4.3. FTIR Spectroscopy

FTIR spectra were recorded with a Nicolet iS10 spectrometer, Thermo Fisher Scientific, Waltham city, MA, USA. Dried sample powder was thoroughly mixed with KBr and pressed into pellets. Measurements covered 400–4000 cm^−1^ at a resolution of 1.0 cm^−1^.

#### 2.4.4. TG-DSC

Thermal behavior was examined with a NETZSCH STA 449C simultaneous TG-DSC analyzer, NETZSCH, Selb city, Germany. Approximately 20 mg of powder was heated from 30 to 1000 °C at 10 °C/min under nitrogen. Mass losses in the relevant temperature intervals were used to estimate the contents of thermally decomposing phases.

#### 2.4.5. SEM and EDS

The microstructure and morphology of the samples were characterized using a scanning electron microscope (SEM, Gemini 300, Carl Zeiss AG, Oberkochen city, Germany). The hardened cement paste samples at designated curing ages were cut into cubic samples with dimensions of approximately 0.5 cm × 0.5 cm × 0.5 cm using a wire-cutting method. Before SEM observation, the samples were coated with a thin gold layer to enhance electrical conductivity and then mounted on the sample holder using conductive adhesive. After achieving the required vacuum level, SEM images were collected under an accelerating voltage of 20 kV and a working distance of 15.6 mm. The working distance was adjusted according to the selected magnification.

## 3. Results and Discussion

### 3.1. Determination of Ultrasonication Time for Nano-TiO_2_ Suspension

**Figure 3:**
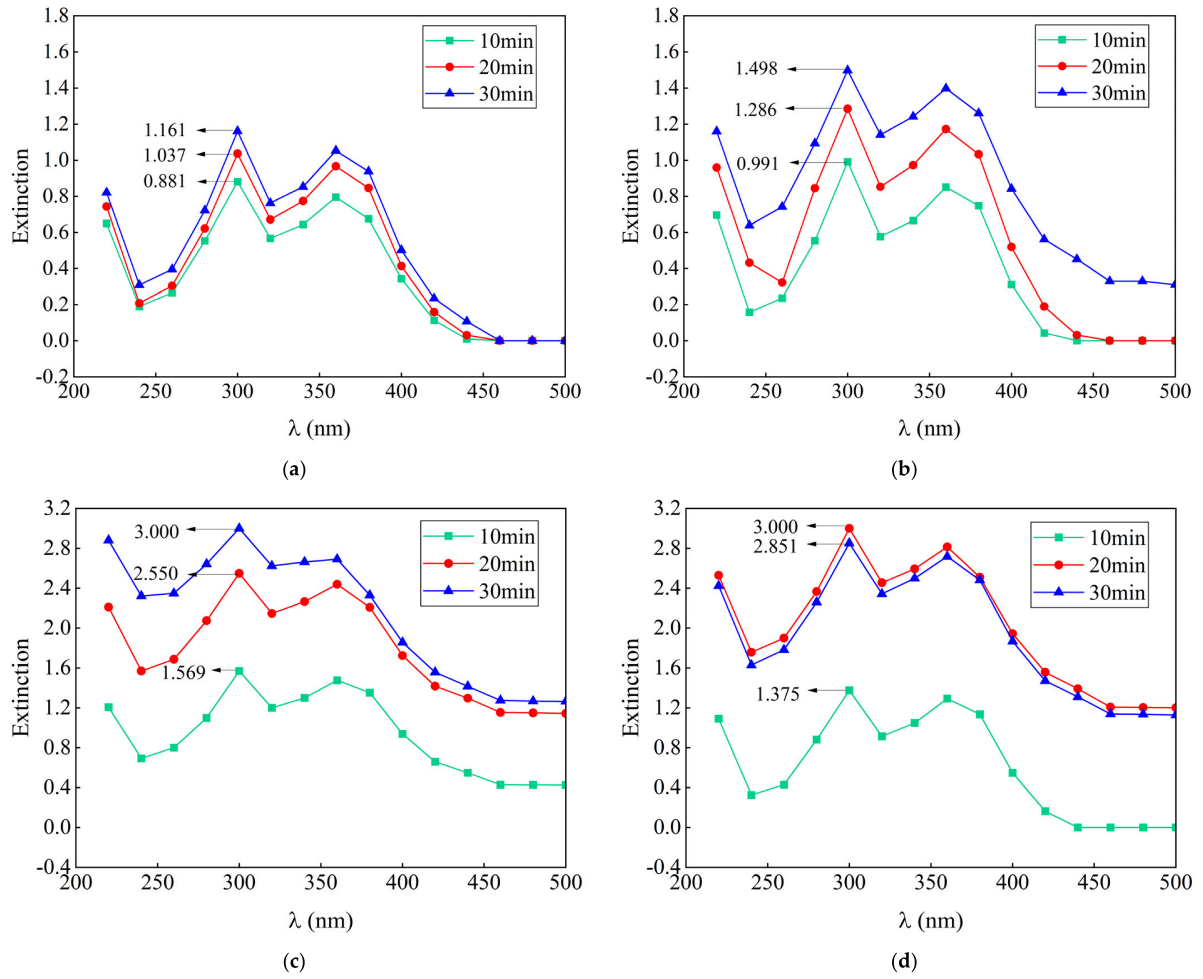
The spectrophotometric results of nano-TiO_2_ suspension: (**a**) 3%; (**b**) 6%; (**c**) 9%; (**d**) 12%.

The results of ultrasonic dispersion for nano-TiO_2_ suspension at different times are shown in Figure 3. As seen in Figure 3a, Figure 3b, and Figure 3c, the extinction of TiO_2_ at 300 nm increased with dispersion time when the nano-TiO_2_ contents were 3%, 6%, and 9%, respectively. It should be noted that for dispersions of micrometer-sized agglomerates, light scattering significantly contributes to the attenuation of radiation intensity. Hence, this measurement reflected total optical extinction rather than pure molecular absorption. In Figure 3d, with a nano-TiO_2_ content of 12%, the extinction at 300 nm increased as ultrasonication time extended from 10 min to 20 min. However, when ultrasonication time further increased from 20 min to 30 min, the extinction value decreased from 3.000 to 2.851. This critical stabilization and subsequent reversal behavior thoroughly aligned with recent findings in international literature regarding nanoparticle dispersion dynamics. As comprehensively reviewed by McCormack et al. [54], the dispersion protocol, specifically the duration and power of sonication, fundamentally dictated the physicochemical identity and agglomeration state of nano-TiO_2_ particles. While initial ultrasonic cavitation imparted high shear energy to break up large agglomerates, extending the sonication time beyond an optimal threshold introduces excessive thermal and mechanical energy. This phenomenon caused a steep rise in suspension temperature and dramatically accelerated the Brownian motion of the nano-TiO_2_ particles, thereby increasing their collision frequency. Concurrently, excessive acoustic energy can disrupt the adsorption equilibrium of the water-reducing agent on the nanoparticle surfaces. Driven by strong van der Waals forces, these destabilized particles inevitably underwent severe secondary re-agglomeration, causing the optical extinction to decrease. This mechanism was further supported by Kim et al. [55], who demonstrated that optimizing ultrasonication time was mandatory to control the colloidal aggregate size of TiO_2_, which directly governed its photocatalytic efficiency. Our finding successfully contextualized this generic nanoscale law within the specific design of cementitious matrices, clarifying the boundaries for optimal dispersion protocols. Therefore, to maintain consistent dispersion conditions across all nano-TiO_2_ concentrations and achieve optimal dispersion effects, the ultrasonication time for the nano-TiO_2_ suspension in this study was set at 20 min.

### 3.2. Compressive Strength of Cement Paste

**Figure 4:**
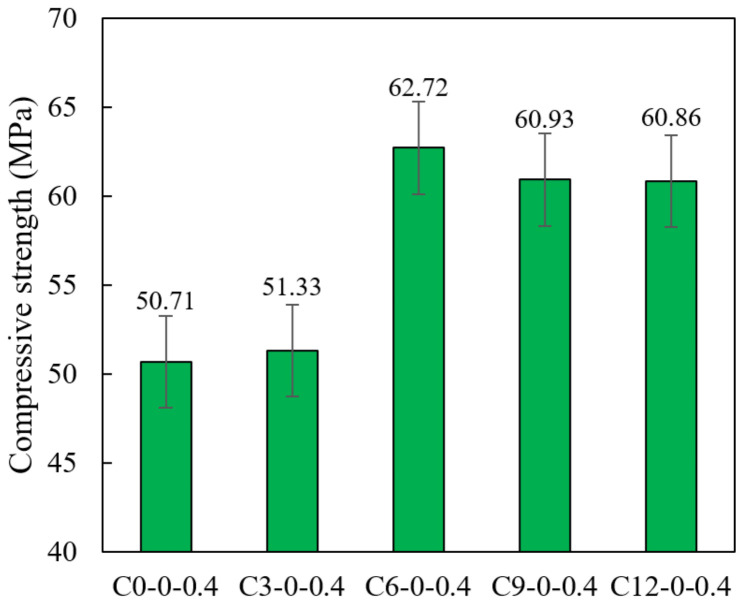
Effect of nano-TiO_2_ content on the compressive strength of cement paste.

The 28 d strengths of C0-0-0.4, C3-0-0.4, C6-0-0.4, C9-0-0.4, and C12-0-0.4 are presented in Figure 4. The control reached 50.71 MPa, while the mixtures containing 3%, 6%, 9%, and 12% nano-TiO_2_ reached 51.33, 62.72, 60.93, and 60.86 MPa, respectively. Relative to the control, these values correspond to increases of 1.22%, 23.7%, 20.2%, and 20.0%. Strength therefore increased up to a nano-TiO_2_ dosage of 6% and declined slightly at higher contents. Although the 9% and 12% mixtures remained stronger than the control and 3% mixture, neither exceeded the 6% result.

At a suitable dosage, nano-TiO_2_ provides additional nucleation surfaces for hydration and fills small voids, thereby refining the matrix and increasing strength [56]. When the dosage becomes excessive, however, particle agglomeration and local interference with crystal growth can create a less uniform pore structure. The reduction in strength above 6% is consistent with this transition from effective nucleation and microfilling to agglomeration-dominated behavior.

### 3.3. Effect of Nano-TiO_2_ Content on NO Gas Photocatalytic Degradation of Cement Paste

**Figure 5:**
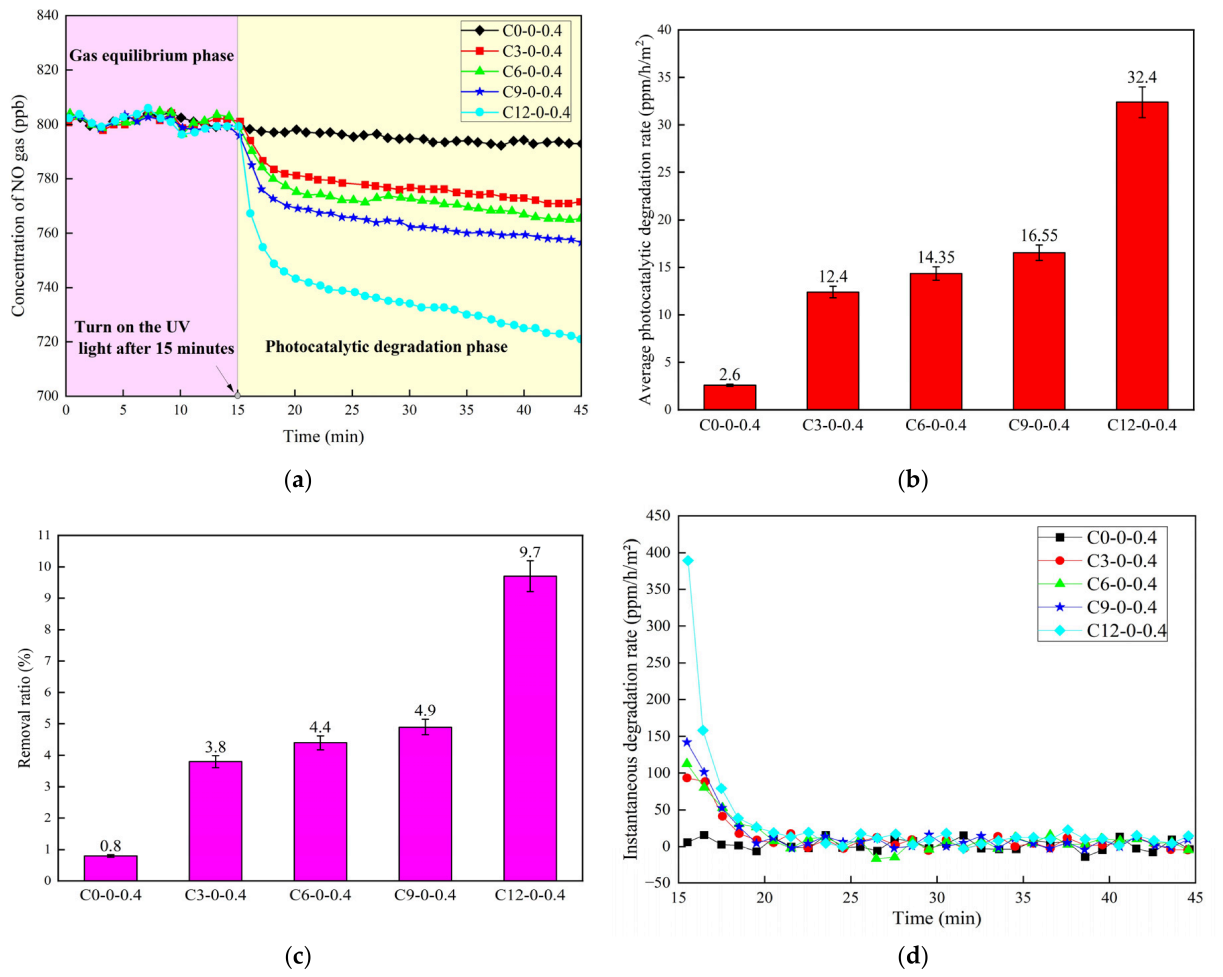
Effect of nano-TiO_2_ content on NO gas photocatalytic degradation of cement paste: (**a**) Concentration of NO gas; (**b**) Average photocatalytic degradation rate of NO gas; (**c**) Removal ratio of NO gas; (**d**) Instantaneous photocatalytic degradation rate of NO gas.

Figure 5 illustrates the effect of nano-TiO_2_ content (0%, 3%, 6%, 9%, and 12%) on the photocatalytic degradation of NO gas by the cement paste. Figure 5a shows the variation process of the NO gas concentration. It can be observed that as the reaction time increased, the control group exhibited a relatively low adsorption of the NO gas, while the samples containing nano-TiO_2_ consistently reduced the NO gas concentration. Figure 5b and Figure 5c presented the average photocatalytic degradation rate and removal ratio of the NO gas, respectively, for different nano-TiO_2_ contents. Both the average photocatalytic degradation rate and removal ratio increased with higher nano-TiO_2_ content. The removal ratio showed a gradual increase between 3% and 9% of the nano-TiO_2_ content. At a nano-TiO_2_ content of 12%, the removal ratio reached 9.73%, indicating enhanced photocatalytic activity with increasing nano-TiO_2_ content. Although the maximum NO-removal ratio obtained in this study was below 10%, its practical significance should be evaluated in relation to the incorporation method and test conditions. Commercial and field-applied photocatalytic cementitious systems are commonly designed to maximize the exposure of TiO_2_ at the material surface. For example, a photocatalytic cement-based coating applied in the Umberto I tunnel in Rome produced an NOx reduction of approximately 20% based on a direct comparison before and after renovation [68]. A commercial porous photocatalytic coating, Protectam FN2 containing P25 TiO_2_, achieved an NO-abatement rate of up to 75 μmol m^−2^ h^−1^ at an inlet concentration of 0.1 ppmv, a gas-flow rate of 3000 cm^3^ min^−1^, and a relative humidity of 50% [69]. Commercial TX Active-based paving blocks have also been manufactured with the photocatalytic material concentrated in a 5 mm surface layer to improve light exposure and pollutant contact [70]. However, these values cannot be directly compared with the present removal ratio because the reported systems differ substantially in photocatalyst location, exposed surface area, pollutant concentration, gas-flow rate, relative humidity, irradiance, reactor geometry, and performance metric. In contrast, the present study employed bulk-incorporated nano-TiO_2_ in cement paste, in which a considerable proportion of the photocatalyst was embedded within hydration products and was therefore less accessible to UV irradiation and gaseous NO. Accordingly, the removal ratio of 9.73% represents measurable photocatalytic activity achieved within a structurally integrated cementitious matrix, while also reflecting the trade-off between photocatalyst accessibility and mechanical performance.

Figure 5d shows the instantaneous photocatalytic degradation rate of the NO gas, representing the degradation rate at each reaction moment. The results revealed that the degradation rate peaked at the onset of the reaction, with the sample containing 12% nano-TiO_2_ and achieving a degradation rate of 389.3 ppm/h/m^2^. Subsequently, the degradation rates of all samples rapidly decreased within 5 min and remained basically constant thereafter, indicating that the majority of the reaction occurred within the first 5 min of the 30 min test period. This observed kinetic behavior-characterized by an initial rapid degradation stage followed by a sharp decline-was generally consistent with the photocatalytic reaction characteristics reported for TiO_2_-incorporated cementitious materials in recent years. Similar trends have been summarized in recent reviews on nano-TiO_2_ cement-based photocatalytic materials, where the degradation efficiency was typically highest during the initial irradiation stage due to the abundance of exposed active sites and rapid surface adsorption of NO molecules [3,38]. As the reaction proceeded, the accumulation of intermediate oxidation products and the gradual occupation of active sites can significantly suppress the subsequent photocatalytic reaction rate. Meanwhile, in bulk-incorporated cementitious matrices, many nano-TiO_2_ particles remained embedded within the hydration products and were therefore less accessible to UV irradiation and gaseous pollutants, resulting in diffusion-limited reaction kinetics.

Furthermore, previous studies have demonstrated that excessive TiO_2_ incorporation may lead to nanoparticle agglomeration and shielding effects, thereby limiting the effective utilization of photocatalytically active surfaces [38]. Therefore, although increasing the nano-TiO_2_ content generally improved the NO removal efficiency in this study, the enhancement tendency gradually weakened at higher dosages.

For direct comparison with recent international studies, the maximum NO removal ratio obtained in this study (9.73%) fell within the commonly reported range for bulk-incorporated TiO_2_ cementitious materials under UV irradiation. Recent reviews and experimental studies on photocatalytic cement-based materials have reported NOx removal efficiencies generally ranging from approximately 5% to 15%, depending on TiO_2_ dosage, light intensity, porosity, and exposure conditions [38,57]. The relatively moderate removal efficiency observed in this work may be attributed to the inherent mass-transfer limitation and partial encapsulation of nano-TiO_2_ particles within the dense cement hydration matrix, which restricts the accessibility of active photocatalytic sites.

Regarding the fate of NO and NO_2_ conversion dynamics, although the current experimental setup (single-gas analyzer) limited the dynamic monitoring of transient gas-phase NO_2_, which posed a constraint on achieving a fully closed-loop gas-phase NOx mass balance, the solid-state transformation pathway offered crucial insights. Mechanistically, the photocatalytic oxidation of NO inevitably proceeded via NO_2_ as a critical intermediate. On inert or acidic substrates, the rapid desorption of this toxic intermediate often resulted in secondary pollution [6]. However, within the highly alkaline hydration environment of the cement paste, the abundant Ca(OH)_2_ crystals served as an immediate in situ chemical trap. The transient NO_2_ intermediates generated on the nano-TiO_2_ active sites underwent rapid acid-base neutralization before they could desorb and escape into the gas stream, transforming into stable solid-state calcium nitrate Ca(NO_3_)_2_ [49]. This immobilized chemical fate was strongly cross-verified by the subsequent microstructural evidence in Section 3.7 and Section 3.9, where crystalline Ca(NO_3_)_2_·2H_2_O was successfully indexed via surface-enriched XRD analysis. Therefore, while dynamic gas-phase tracking was restricted by equipment boundaries, the solid-state product accumulation provided a reliable time-integrated validation that the measured NO depletion corresponds to genuine chemical immobilization rather than a mere conversion into escaped NO_2_ gas. Future investigations will incorporate multi-gas chemiluminescence detectors to map the transient gaseous NO_x_ balance with higher temporal resolution.

It should be noted that the current study did not monitor gas-phase NO_2_ concentration. Thus, a full NOx mass balance could not be established. Future work will employ multi-gas analyzers to quantify the transient NO and NO_2_ dynamics.

To further clarify the photocatalytic degradation mechanism and evaluate the long-term environmental safety of nano-TiO_2_ cementitious materials, the adsorption behavior of NO gas and the immobilization stability of nano-TiO_2_ particles within the cement matrix were further discussed. The hydrated cement paste naturally possessed a highly porous network with a large internal surface area, which provided abundant initial physical adsorption sites for gaseous NO molecules, facilitating their localized concentration and subsequent mass transport to the photo-active titanium centers. Regarding the long-term stability and safety of the nanomaterials, because the nano-TiO_2_ particles were bulk-incorporated during the mixing phase, they became thoroughly encapsulated and mechanically locked within the dense, amorphous networks of the calcium silicate hydrate (C–S–H) gel [58]. Recent accelerated weathering and leaching studies on nano-engineered concrete demonstrated that the cement matrix encapsulation efficiency remained exceptionally high, successfully suppressing the secondary release of nanoparticles into the run-off water or ambient air during its conventional service life [59,60]. Therefore, the bulk immobilization strategy contributed to both sustained photocatalytic durability and reduced environmental release risk of nano-TiO_2_ particles. The present findings were generally consistent with recent studies on nano-TiO_2_ cementitious materials, which reported that bulk incorporation of nano-TiO_2_ can effectively maintain photocatalytic durability while minimizing nanoparticle release risks during service conditions [59,61].

#### Apparent Kinetic Analysis

To evaluate the apparent kinetic behavior of photocatalytic NO removal, the concentration–time data recorded after UV irradiation were fitted using pseudo-first-order and pseudo-second-order kinetic models. Because the experiments were conducted in a continuous-flow reactor and the outlet NO concentration approached a non-zero steady-state value, the following models were adopted:

(1) $$C_{t}=C_{ss}+(C_{0}-C_{ss})\exp(-k_{1}t)$$

(2) $$C_{t}=C_{ss}+(C_{0}-C_{ss})/(1+k_{2}(C_{0}-C_{ss})t)$$

where C_0_, C_t_, and C_ss_ represent the NO concentrations at the beginning of UV irradiation, at irradiation time t, and at the fitted steady state, respectively. k_1_ and k_2_ are the apparent pseudo-first-order and pseudo-second-order rate constants. Model performance was evaluated using the coefficient of determination (R^2^) and root-mean-square error (RMSE). The model with a higher R^2^ and lower RMSE was considered to provide a better description of the experimental data.

The concentration–time data obtained during the initial 5 min after UV irradiation were fitted using apparent pseudo-first-order and pseudo-second-order models. The resulting kinetic parameters are summarized in Table 5.

As shown in Table 5, the apparent pseudo-first-order model yielded R^2^ values of 0.9928, 0.9978, 0.9940, and 0.9995 for C3-0-0.4, C6-0-0.4, C9-0-0.4, and C12-0-0.4, respectively. These values were slightly higher than those obtained using the apparent pseudo-second-order model, while the corresponding RMSE values were lower. Therefore, the initial NO-removal behavior during the first 5 min of UV irradiation was better described by an apparent pseudo-first-order model under the present continuous-flow conditions. It should be emphasized that the fitted kinetic order represents an apparent response under a fixed inlet concentration, gas-flow rate, relative humidity, irradiance, and reactor configuration, rather than an intrinsic reaction order.

### 3.4. Effect of Air-Entraining Agent Content on NO Gas Photocatalytic Degradation of Cement Paste with Nano-TiO_2_

**Figure 6:**
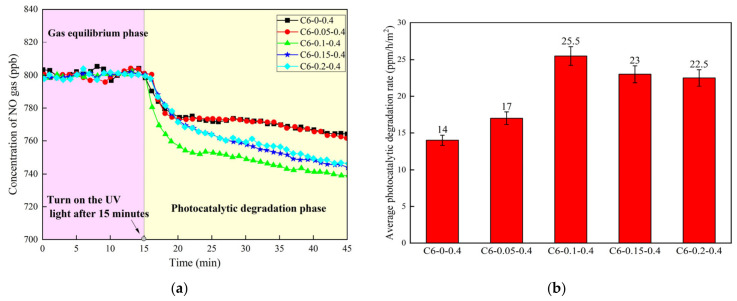

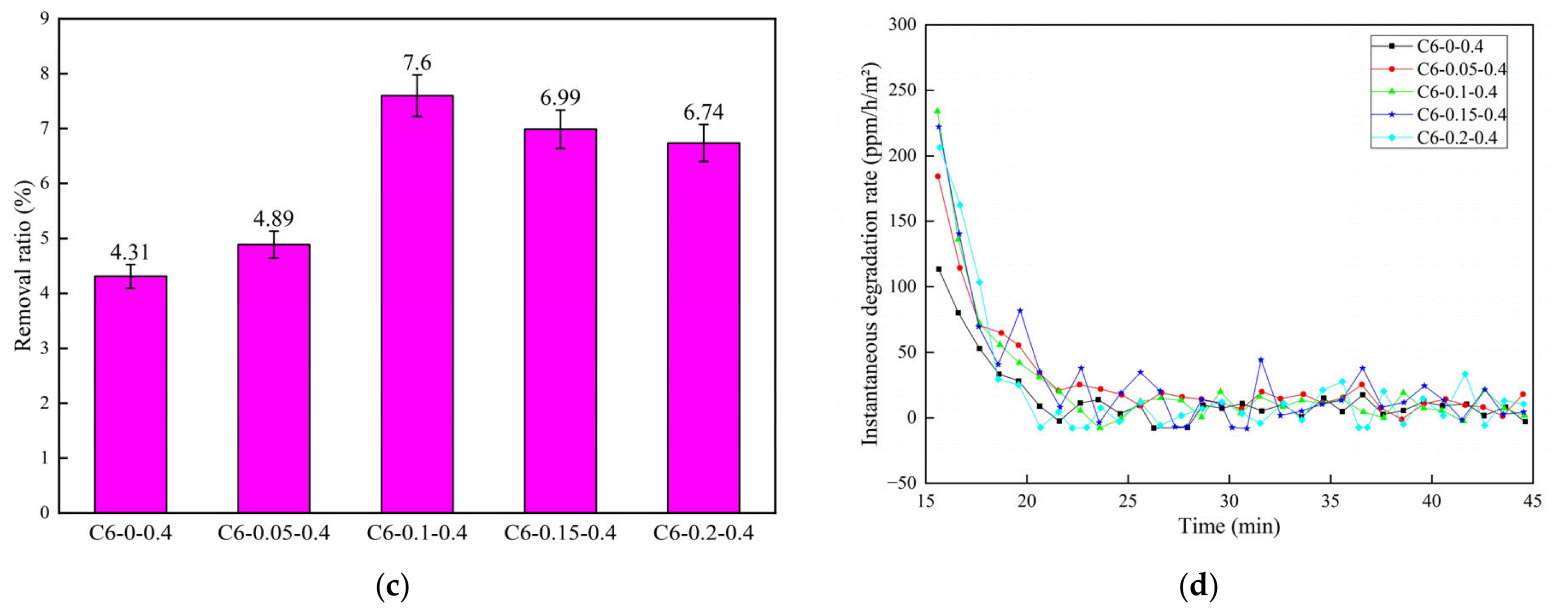
Effect of air entraining agent content on NO gas photocatalytic degradation of cement paste: (**a**) Concentration of NO gas; (**b**) Average photocatalytic degradation rate of NO gas; (**c**) Removal ratio of NO gas; (**d**) Instantaneous photocatalytic degradation rate of NO gas.

Figure 6 presents the effect of air-entraining agent (AEA) dosage on the photocatalytic degradation performance of NO gas by nano-TiO_2_ cement paste. The results demonstrated that the photocatalytic degradation efficiency first increased and then decreased with increasing AEA dosage, indicating the existence of an optimal air-entraining agent content. The sample containing 0.10% AEA exhibited the highest NO removal efficiency. Similar non-monotonic trends have also been reported in recent studies on photocatalytic cementitious materials containing surfactant-based admixtures [38]. The enhancement in photocatalytic performance at low AEA dosage can be mainly attributed to the improvement of pore connectivity and internal gas diffusion pathways within the cementitious matrix. The incorporation of a moderate amount of air-entraining agent increased the porosity and promoted the transport of NO molecules toward the photocatalytically active TiO_2_ surfaces, thereby improving the contact probability between pollutants and active sites. Similar effects of pore structure optimization on photocatalytic efficiency have been reported in previous studies on TiO_2_-modified cementitious materials [61]. However, when the AEA dosage further increased, the photocatalytic degradation efficiency gradually declined. This phenomenon may be associated with the excessive incorporation of surfactant molecules, which can partially encapsulate TiO_2_ particles and hydration products, thereby hindering UV light penetration and decreasing the accessibility of photocatalytically active surfaces. In addition, excessive porosity may adversely affect the compactness of the cementitious matrix and reduce the effective surface exposure of TiO_2_ particles. These results indicated that the photocatalytic performance of nano-TiO_2_ cement paste was governed by a competitive relationship between enhanced gaseous diffusion pathways and surfactant-induced surface shielding effects.

### 3.5. Effect of Water-Cement Ratio on NO Gas Photocatalytic Degradation of Cement Paste with Nano-TiO_2_

**Figure 7:**
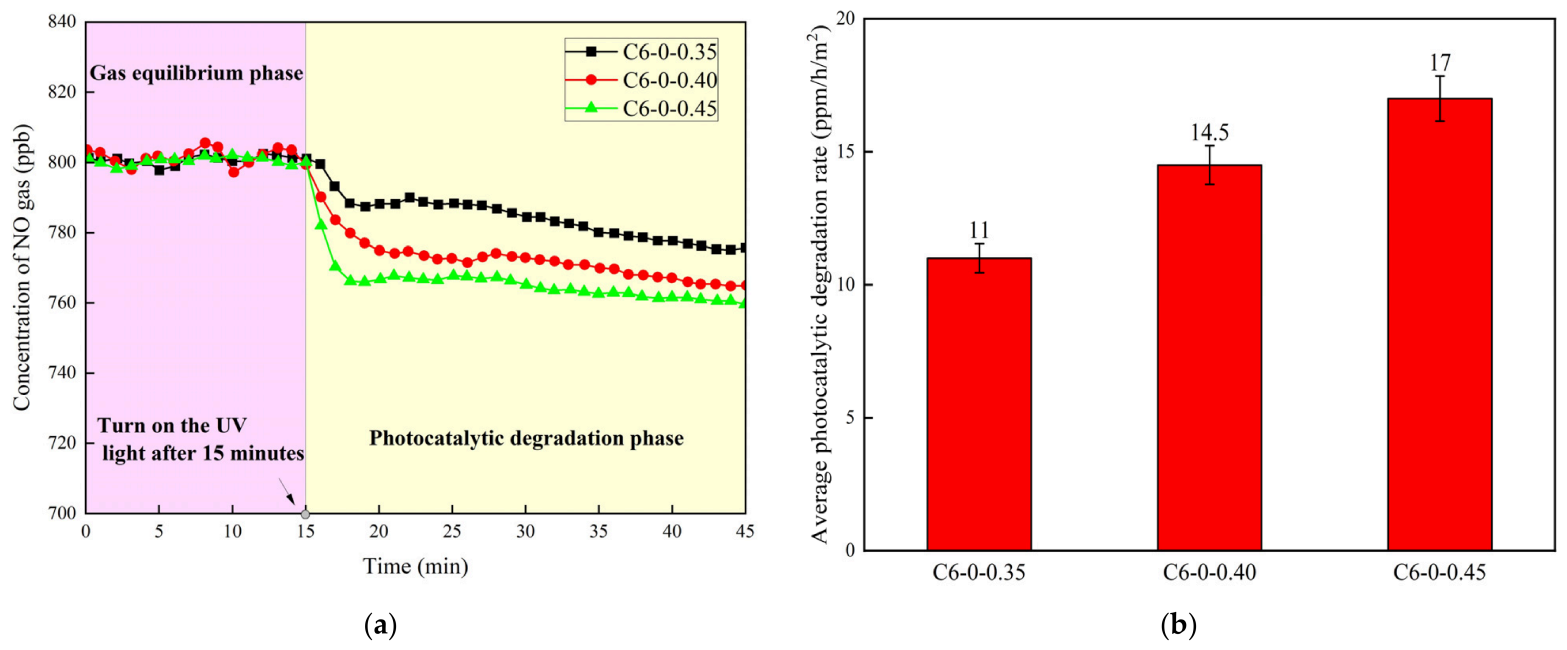

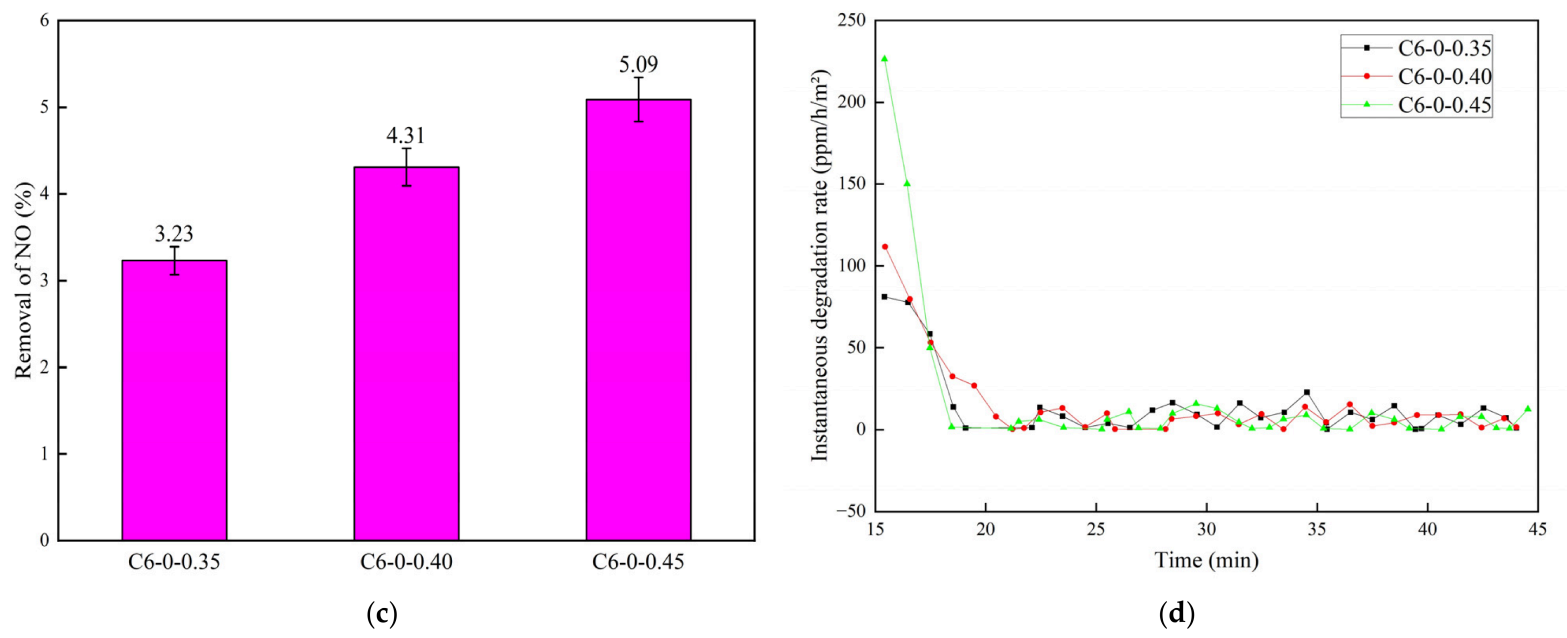
Effect of water-to-cement ratio on NO gas photocatalytic degradation of cement paste: (**a**) Concentration of NO gas; (**b**) Average photocatalytic degradation rate of NO gas; (**c**) Removal ratio of NO gas; (**d**) Instantaneous photocatalytic degradation rate of NO gas.

Figure 7 shows the influence of water-cement ratio on the photocatalytic degradation performance of NO gas. As the water-cement ratio increased, both the average photocatalytic degradation rate and NO removal ratio gradually increased. Similar effects of pore structure enhancement induced by increased water-cement ratio have also been reported in recent studies on photocatalytic cement-based materials [57]. The increase in water-cement ratio generally led to higher porosity and a more interconnected pore network within the cement paste, which facilitated the diffusion and adsorption of NO molecules. Improved gas transport conditions enhanced the accessibility of pollutants to photocatalytically active TiO_2_ surfaces, thereby promoting photocatalytic oxidation reactions. Previous studies have demonstrated that pore structure played a critical role in determining the photocatalytic efficiency of TiO_2_ cementitious materials, especially for gaseous pollutant removal applications. However, although a higher water-cement ratio was beneficial for pollutant diffusion and photocatalytic activity, excessively high porosity may negatively affect the compactness, mechanical integrity, and long-term durability of the cementitious matrix [57]. Therefore, the optimization of water-cement ratio should balance photocatalytic performance and material durability requirements.

### 3.6. Effect of Curing Age on NO Gas Photocatalytic Degradation of Cement Paste with Nano-TiO_2_

**Figure 8:**
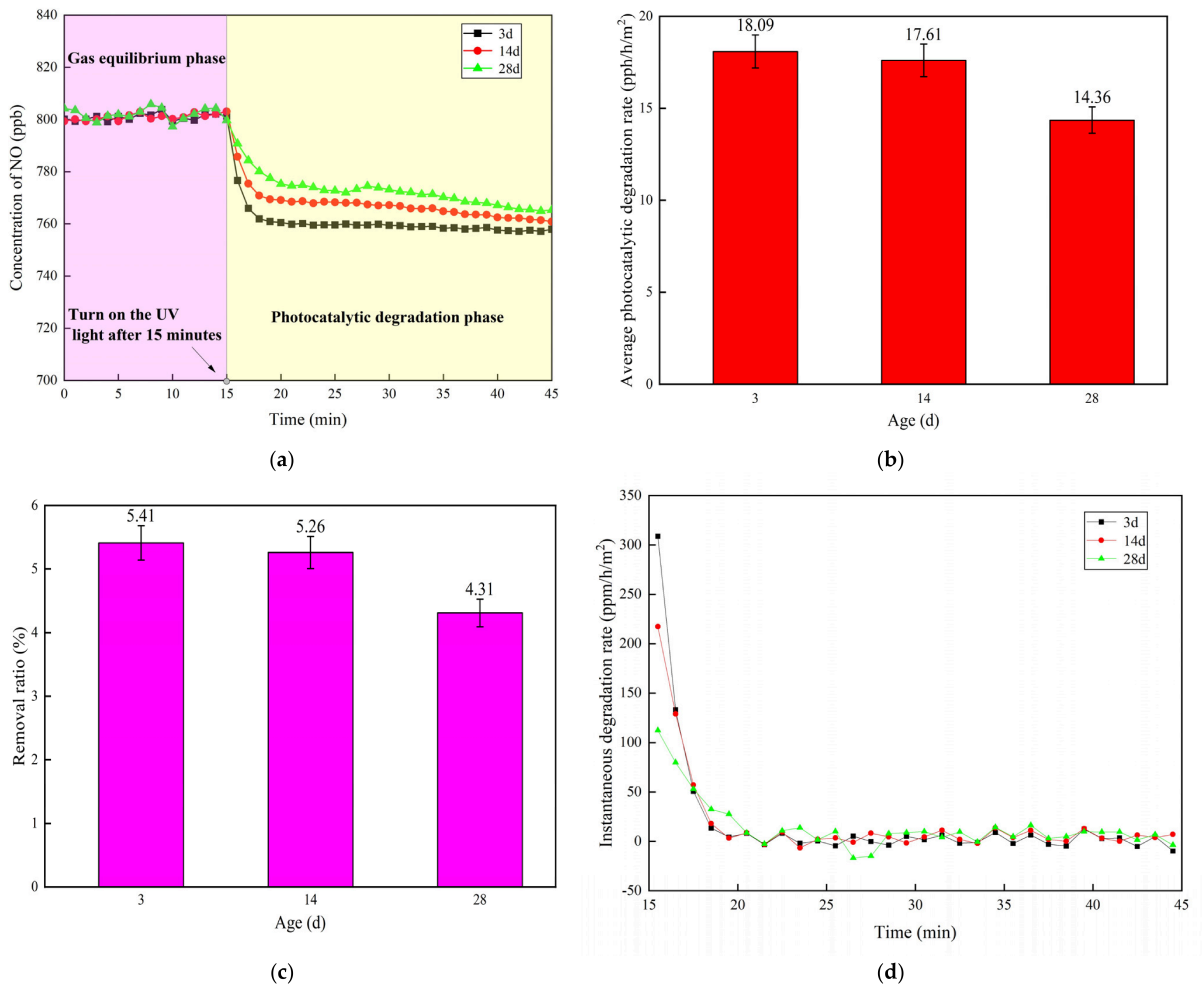
Effect of curing age on NO gas photocatalytic degradation of cement paste: (**a**) Concentration of NO gas; (**b**) Average photocatalytic degradation rate of NO gas; (**c**) Removal ratio of NO gas; (**d**) Instantaneous photocatalytic degradation rate of NO gas.

The effect of curing age is presented in Figure 8. Both the average degradation rate and the NO removal ratio decreased as curing proceeded. Comparable age-dependent changes have been reported for TiO_2_-containing cementitious materials [38,58]. The reduction in photocatalytic activity with increasing curing age was mainly associated with the progressive hydration process of cement paste. As hydration proceeds, larger amounts of hydration products such as calcium silicate hydrate (C–S–H) gel and calcium hydroxide were continuously generated, resulting in a denser microstructure. Although matrix densification improved the mechanical stability and compactness of the cementitious material, the hydration products may progressively cover part of the exposed TiO_2_ active sites and reduce UV accessibility, thereby decreasing photocatalytic efficiency [58]. In addition, the denser hydration structure formed at later curing ages may hinder the diffusion and adsorption of gaseous NO molecules within the cementitious matrix, leading to mass-transfer limitations during the photocatalytic process. Similar mechanisms have also been discussed in recent studies on nano-TiO_2_ cement-based photocatalytic materials, where the balance between matrix densification and active-site exposure was considered a key factor affecting long-term photocatalytic performance [38].

In addition to the influence of curing age, the long-term photocatalytic performance of nano-TiO_2_-modified cementitious materials may be affected by environmental exposure conditions during service. Carbonation, caused by reactions between CO_2_ and cement hydration products, may modify the chemical composition and pore structure of the cement matrix, thereby influencing NO transport pathways and the accessibility of nano-TiO_2_ active sites. Meanwhile, weathering processes such as wetting–drying cycles and ultraviolet exposure may gradually alter surface morphology and microstructure, resulting in changes in photocatalytic efficiency. Mechanical abrasion during service may also influence photocatalytic durability by modifying the exposed surface area of TiO_2_ particles.

Considering the decrease in photocatalytic activity with increasing curing age observed in this study, appropriate surface activation strategies may provide potential approaches for maintaining photocatalytic performance during long-term service. Controlled polishing or mild surface treatment could remove part of the surface hydration products and expose previously embedded nano-TiO_2_ particles, thereby increasing the availability of photocatalytic active sites. However, excessive surface removal may cause TiO_2_ loss and compromise durability. Therefore, future studies should focus on optimizing surface renewal strategies to achieve a balance between photocatalytic activity recovery and structural stability.

To further clarify the relationship between mixture parameters, pore structure, and photocatalytic performance, the MIP and N_2_ adsorption results were considered together with the NO-removal results. For the samples containing 0%, 6%, and 12% nano-TiO_2_, the MIP-accessible porosity initially decreased and then increased with increasing nano-TiO_2_ dosage. The sample containing 6% nano-TiO_2_ exhibited the lowest porosity of 16.1%, whereas the porosity increased to 17.6% at a nano-TiO_2_ dosage of 12%, accompanied by an increase in the macropore fraction to 8.68%. The cumulative nanopore volumes obtained from N_2_ adsorption for the specimens containing 0%, 6%, and 12% nano-TiO_2_ were 0.043, 0.036, and 0.040 cm^3^/g, respectively [71]. These results indicated that an appropriate nano-TiO_2_ dosage promoted pore refinement through nucleation and microfilling effects, which was consistent with the highest compressive strength obtained at 6% nano-TiO_2_. However, the maximum NO-removal ratio was obtained at 12% nano-TiO_2_, demonstrating that photocatalytic performance was not controlled by porosity alone. The higher photocatalyst dosage provided more potential active sites, while the increased macropore fraction may have reduced part of the resistance to gaseous NO transport. At the same time, nanoparticle agglomeration and encapsulation by hydration products may have limited the effective utilization of the additional nano-TiO_2_.

For the air-entraining-agent series, the MIP results showed that the accessible porosity increased from 16.1% to 17.5%, 22.6%, 29.7%, and 30.7% as the AEA dosage increased from 0% to 0.05%, 0.10%, 0.15%, and 0.20%, respectively. Increasing the AEA dosage generally decreased the proportions of gel and transitional pores and increased the capillary- and macropore fractions [71]. Nevertheless, the highest NO-removal ratio was obtained at an intermediate AEA dosage of 0.10%, rather than at the highest measured porosity. This finding indicated that an increase in accessible porosity alone did not continuously improve photocatalytic performance. Moderate air entrainment may facilitate gaseous NO transport toward exposed TiO_2_ surfaces, whereas excessive AEA addition may generate an overly coarse pore structure and increase the shielding of photocatalytically active surfaces by surfactant molecules. Therefore, the optimum AEA dosage reflected a balance between pore-related gas transport and photocatalyst accessibility.

Sugrañez et al. [15] reported that changes in water-to-cement ratio and mortar composition altered overall porosity and pore-size distribution, and that a greater volume of macropores, defined in their study as pores larger than 80 nm, increased the accessibility of reactants to photocatalytically active sites and enhanced NOx degradation. Accordingly, the increase in NO-removal performance observed with increasing water-to-cement ratio in the present study may be associated with less restricted pore-related gas transport and an increased probability of NO molecules reaching exposed TiO_2_ surfaces. However, an excessively high water-to-cement ratio may compromise matrix compactness and mechanical durability.

Regarding curing age, Chen and Poon [72] found that the NOx-removal ability of TiO_2_-modified cement paste decreased as curing progressed because continued hydration filled capillary pores and formed barriers to both gaseous reactants and incident photons. In the present study, the reduction in NO removal with curing age can therefore be attributed to the combined effects of progressive matrix densification, more restricted pollutant transport, and partial coverage of TiO_2_ active sites by hydration products. The subsequent XRD, FTIR, TG-DSC, and SEM results also supported the progressive development and accumulation of hydration products. Overall, the combined results indicated that photocatalytic NO removal was governed by the interaction among photocatalyst dosage, accessible pore structure, gas transport, and active-site exposure, rather than by total porosity alone.

### 3.7. Analysis of XRD

**Figure 9:**
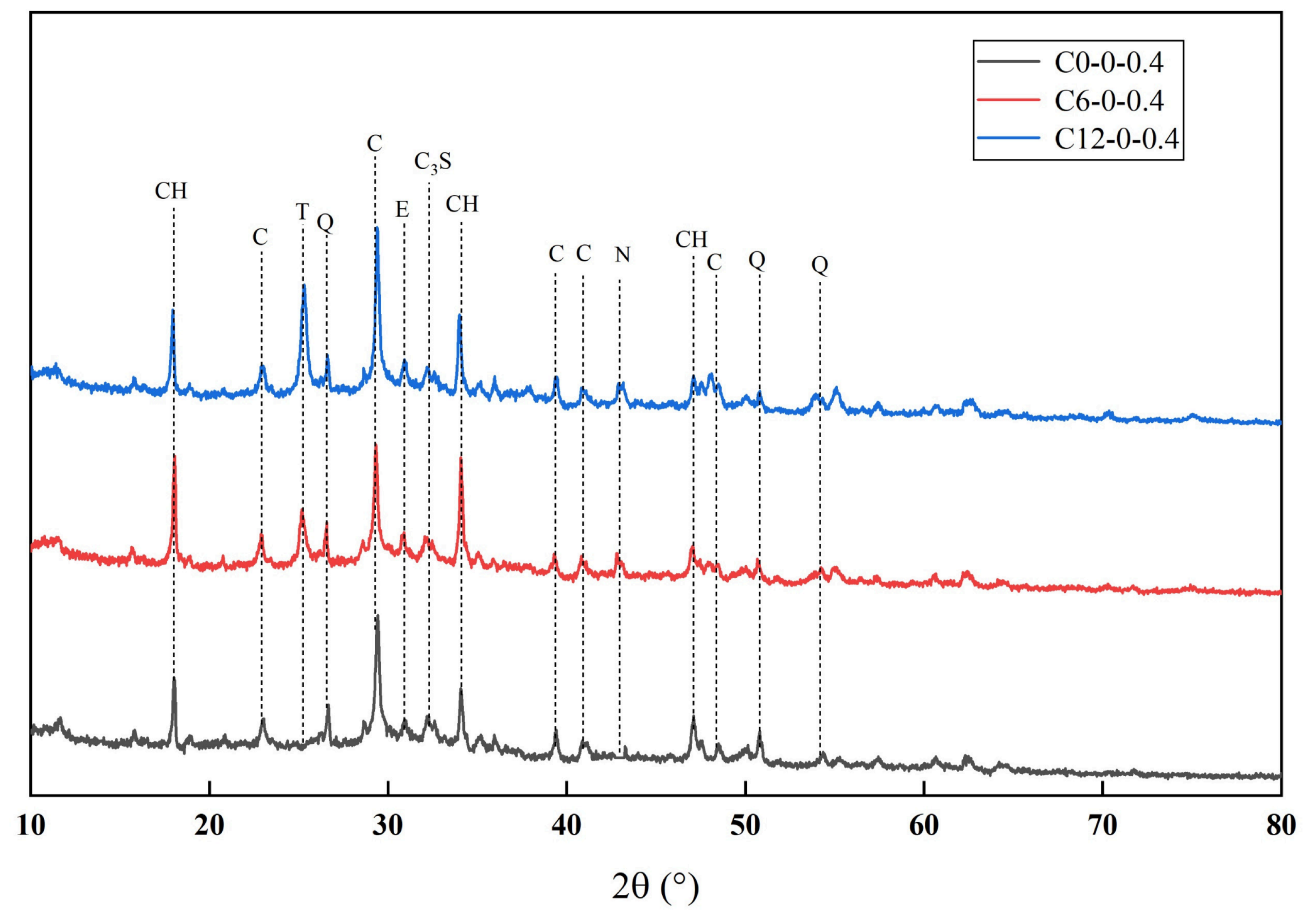
The XRD results of NO gas photocatalytic degradation of cement paste with different contents of Nano-TiO_2_ (CH: Ca(OH)_2_; C: CaCO_3_; T: Nano-TiO_2_; Q: Quartz; E: Ettringite; C_3_S: Tricalcium silicate; N: Ca(NO_3_)_2_·2H_2_O).

The XRD analysis was conducted on the compositions of C0-0-0.4, C6-0-0.4, and C12-0-0.4 samples after the NO gas photocatalytic degradation, as shown in Figure 9. It can be observed that all three sets of samples contained Ca(OH)_2_, CaCO_3_, Nano-TiO_2_, Quartz, Ettringite, and Tricalcium silicate. As the nano-TiO_2_ content increased, the intensity of the peak characterizing Ca(OH)_2_ also increased, indicating a rise in the relative content of Ca(OH)_2_ [62]. This was because the nano-TiO_2_ promoted cement hydration, increasing the yield of hydration products. Furthermore, compared to the C0-0-0.4, samples containing the nano-TiO_2_ exhibited calcium nitrate dihydrate (Ca(NO_3_)_2_∙2H_2_O). This indicated the system can adsorb NO gas via photocatalytic degradation reaction and oxidize NO into nitrate ions. The presence of CaCO_3_ indicated minor carbonation occurred during the sample preparation and curing.

Crucially, the physicochemical characterization and crystalline phase retention of the incorporated nano-TiO_2_ were directly validated in situ within the hydrated cement matrix by the XRD patterns illustrated in Figure 9. The distinct diffraction peak successfully indexed at approximately 25.3° 2θ corresponded precisely to the (101) lattice plane of the anatase phase, providing solid empirical evidence of its mineralogical structure rather than a mere theoretical assumption [53].

### 3.8. Analysis of FTIR

**Figure 10:**
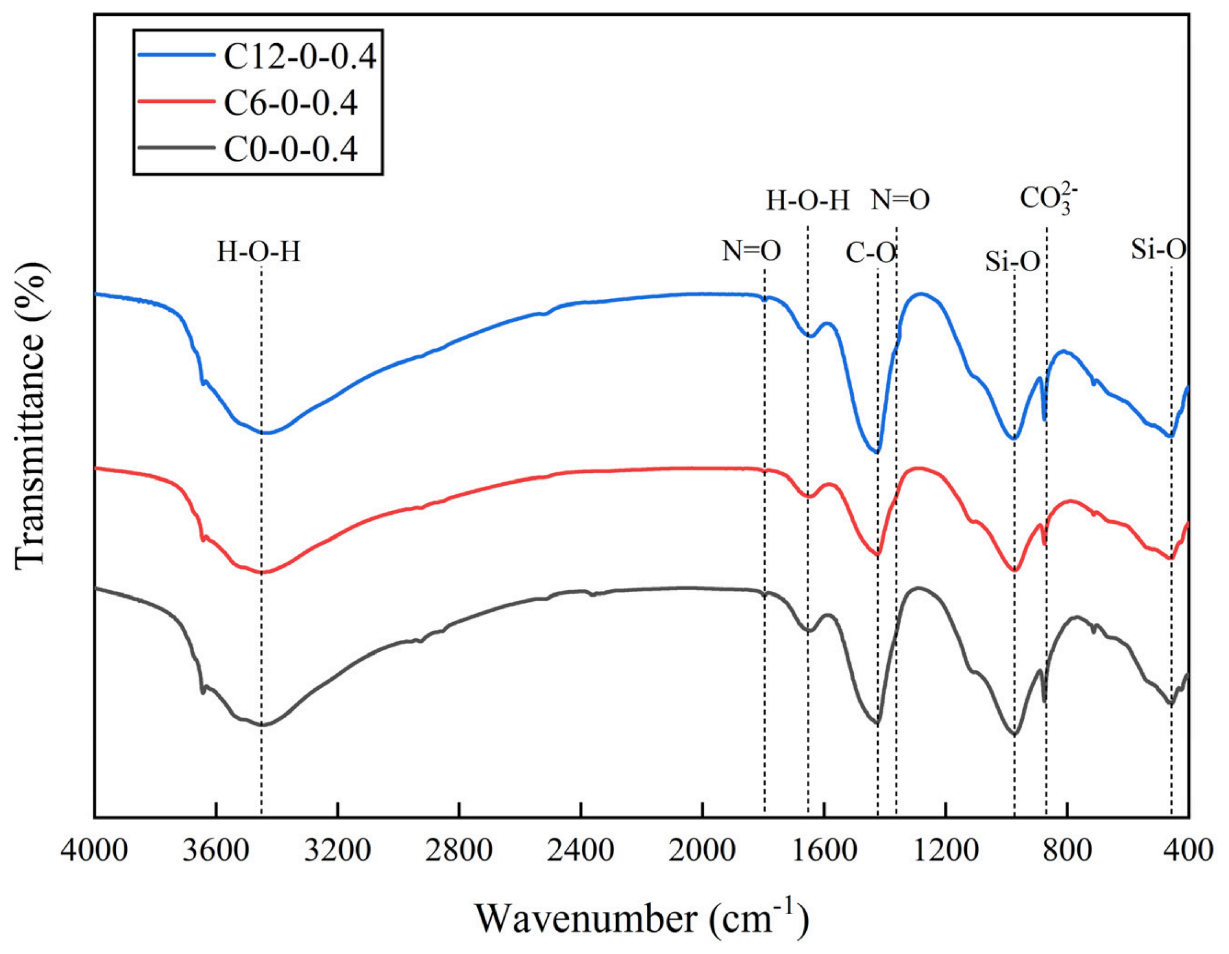
FTIR spectra of cement pastes with different nano-TiO_2_ contents after NO exposure.

FTIR spectra of C0-0-0.4, C6-0-0.4, and C12-0-0.4 after NO exposure are shown in Figure 10. Based on established band assignments [63], the absorption near 460 cm^−1^ corresponds to Si–O bending in silicate tetrahedra, and the band near 970 cm^−1^ is associated with Si–O stretching in Q^2^ units; both are characteristic of C–S–H. The bands at approximately 870 and 1420 cm^−1^ arise from carbonate vibrations and indicate CaCO_3_. Absorbed or interlayer water contributes to the H–O–H bending band near 1650 cm^−1^ and the broad stretching band near 3480 cm^−1^. Thus, C–S–H, carbonate phases, and water were present in all specimens. Literature assignments place N-O-related absorptions in the 1600–1800 cm^−1^ region and nitrate vibrations near 1370 cm^−1^ [64,65,66]. The weak band near 1800 cm^−1^ was associated with adsorbed NO-related species, while the feature at 1370 cm^−1^ in the TiO_2_-containing specimens indicated NO_3_^−^. The control could retain a small quantity of physically adsorbed NO in its surface pores, but it did not show the same nitrate formation. Anatase Ti–O–Ti vibrations generally occur below 800 cm^−1^ and overlap with Si–O bending from C–S–H, so they were not resolved clearly in the FTIR spectra. The presence of anatase was therefore confirmed primarily by XRD. The characteristic Ti–O–Ti vibration bands of anatase TiO_2_ were generally located below 800 cm^−1^. However, these bands overlapped with Si–O bending vibrations originating from C–S–H phases in cementitious materials. Therefore, the TiO_2_-related peaks were not clearly distinguished in the FTIR spectra. The presence and crystalline structure of nano-TiO_2_ were instead confirmed by XRD analysis.

### 3.9. Analysis of TG-DSC

To further clarify the formation mechanism of calcium nitrate during the photocatalytic degradation process, the sequential photo-oxidation and subsequent neutralization reactions were analyzed. Under UV irradiation, the photo-generated reactive radicals (·OH and ·O_2_^−^) on the anatase TiO_2_ surface oxidized gaseous NO into NO_2_ and subsequently into nitric acid (HNO_3_). The generated acidic species further reacted with Ca(OH)_2_ in the cement hydration products, leading to the formation of solid-state calcium nitrate Ca(NO_3_)_2_.

**Figure 11:**
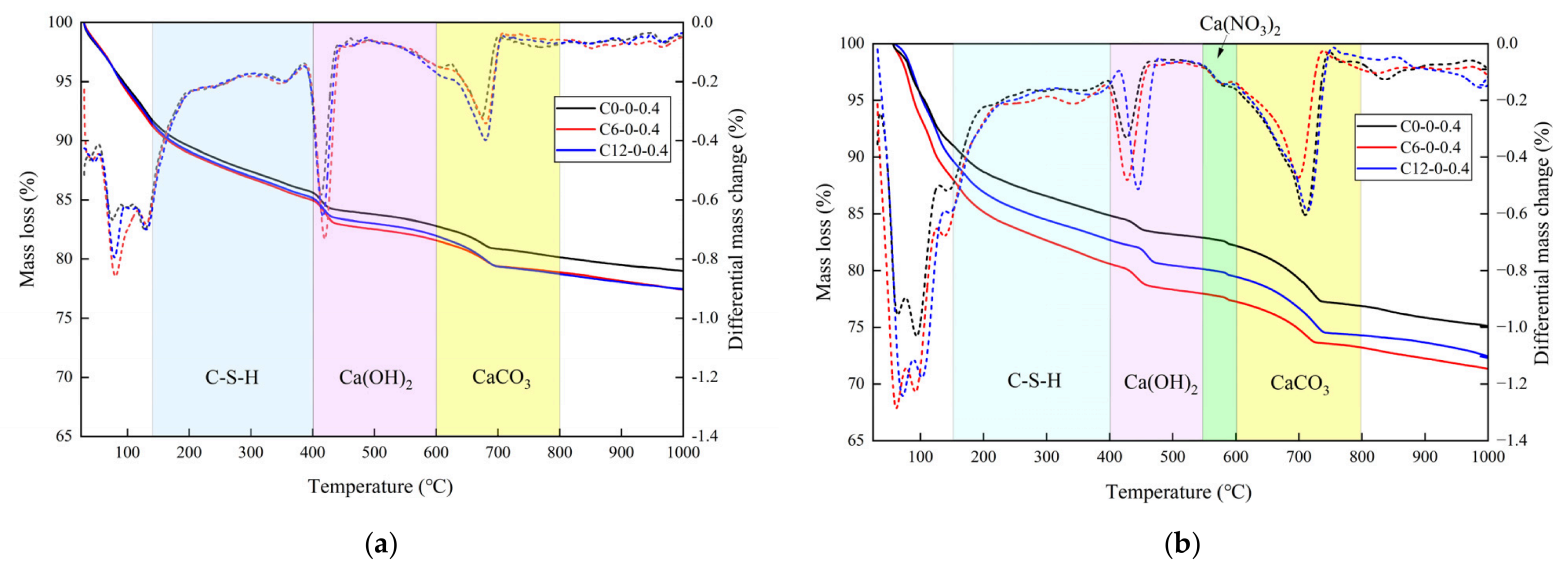
TG curves of cement pastes with different nano-TiO_2_ contents: (**a**) before NO exposure and (**b**) after NO exposure.

To support this reaction pathway, TG-DSC analysis of the cement paste before and after NO gas exposure was comparatively evaluated, as shown in Figure 11a,b. In Figure 11a, the sample before NO exposure exhibited the typical thermal decomposition characteristics of hydrated cement paste, including the dehydration of C–S–H gel at 145–400 °C, the decomposition of Ca(OH)_2_ at 400–600 °C, and the decomposition of CaCO_3_ at 600–800 °C. However, after the photocatalytic NO degradation process, an additional decomposition peak appeared in Figure 11b within the intermediate temperature region, which was absent in the unexposed sample. This newly emerged thermal decomposition feature was attributed to the decomposition of calcium nitrate generated during the photocatalytic reaction process.

Meanwhile, the corresponding TG mass-loss results presented in Table 6 further indicated that the formation trends of Ca(NO_3_)_2_ were closely associated with those of hydration products such as C–S–H gel and Ca(OH)_2_. The sample containing 6% nano-TiO_2_ exhibited the highest formation amount of hydration products and calcium nitrate, which was consistent with the compressive strength development and photocatalytic degradation performance. These results collectively suggested that the photocatalytic oxidation products of NO can be effectively immobilized within the cementitious matrix in the form of calcium nitrate.

### 3.10. SEM

**Figure 12:**
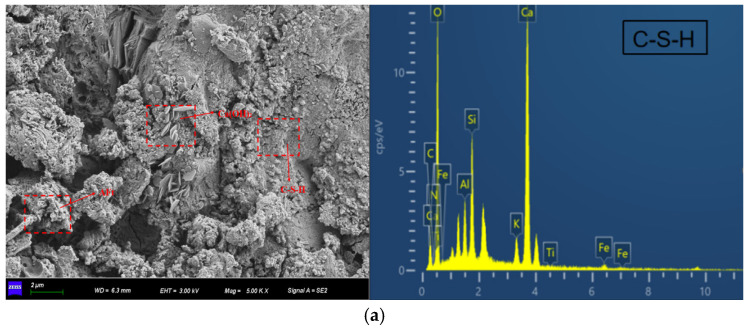

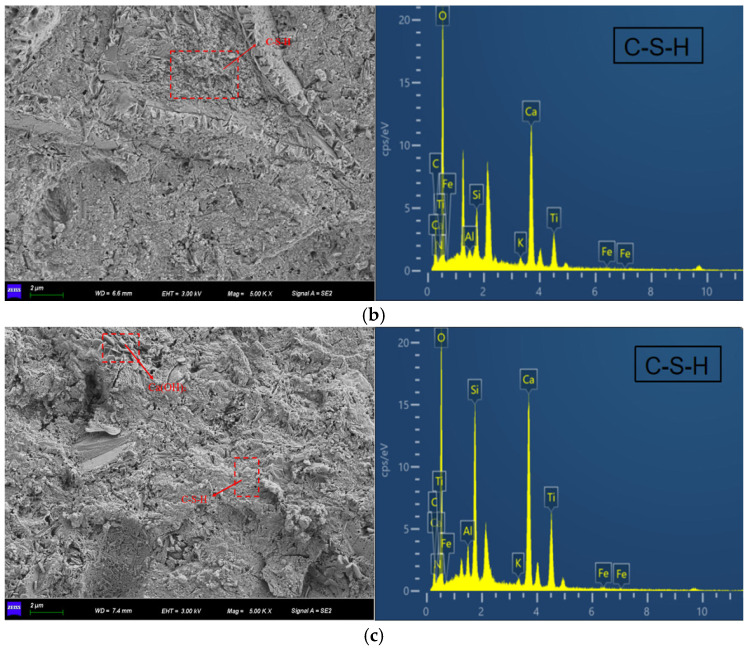
Micro morphology and energy spectrum analysis of photocatalytic cement pastes with different content of Nano-TiO_2_: (**a**) 0% TiO_2_; (**b**) 6% TiO_2_; (**c**) 12% TiO_2_.

The micro-morphology of the cement paste with nano-TiO_2_ dosages of 0%, 6%, and 12% was analyzed using a scanning electron microscope (SEM), and the results are shown in Figure 12. Three main types of hydration products can be observed in the images, including needle-like ettringite, hexagonal plate-like Ca(OH)_2_, and C–S–H gel. This indicates that the incorporation of nano-TiO_2_ did not change the types of hydration products. Comparing the micro-morphologies at different dosages, the structure with a 6% dosage in Figure 12b is the most dense and compact, which therefore exhibited the highest compressive strength.

## 4. Conclusions

This study investigated the effects of nano-TiO_2_ content, air-entraining agent content, water-cement ratio, and curing age on the NO gas photocatalytic degradation of cement paste. The main conclusions are as follows.

1. The compressive strength initially increased and then decreased with increasing nano-TiO_2_ content, peaking at 62.72 MPa at 6% content, which was a 23.7% increase compared with the control group. This occurred because the optimal content of the nano-TiO_2_ can provide nucleation sites to accelerate cement hydration while filling pores.
2. Both the average photocatalytic degradation rate and removal ratio of the NO gas increased with increasing nano-TiO_2_ content. At 12% content, the removal ratio reached 9.73%. With the air-entraining agent content, removal efficiency and average degradation rate initially increased, then decreased, peaking at 7.60% removal efficiency at 0.10% content. As the water-cement ratio increased, both the average photocatalytic degradation rate and removal ratio of the NO gas increased. At a water-cement ratio of 0.45, the removal ratio reached 5.09%, representing a 57.6% increase. With increasing curing age, both the average photocatalytic degradation rate and removal ratio of the NO gas decreased. At 28 d, the removal ratio was 4.31%. The photocatalytic degradation reaction in each group reached its peak rate at the initial moment, with the fastest degradation occurring within the first 5 min. Subsequently, the degradation rate decreased over time, stabilizing after approximately 20 min.
3. Microstructural investigations through surface-enriched XRD analysis verified that the photocatalytic removal of NO gas shifted into a solid-state chemical immobilization pathway within the cementitious matrix. The generated trace nitrogen oxides were trapped in situ by the highly alkaline hydration products (predominantly Ca(OH)_2_, culminating in the accumulation of crystalline calcium nitrate Ca(NO_3_)_2_·2H_2_O. Although a complete dynamic gas-phase NO mass balance was constrained by equipment boundaries, the explicit detection of the solid-state neutralization product confirmed a genuine chemical immobilization fate rather than a mere temporary conversion. It should be noted that the current study did not monitor gas-phase NO_2_ concentration. Thus, a full NOx mass balance could not be established. Future work will employ multi-gas analyzers to quantify the transient NO and NO_2_ dynamics.
4. From the perspective of practical applications, nano-TiO_2_-modified cementitious materials show potential for use in photocatalytic pavements, building façade materials, tunnel linings, and other infrastructure where continuous NOx reduction is required. Compared with surface-coated photocatalytic materials, the incorporation of nano-TiO_2_ into the cement matrix provides better structural stability and reduces the risk of nanoparticle loss during service. However, further studies involving long-term outdoor exposure, durability evaluation, and optimization of photocatalytic activity are required before large-scale engineering applications.

## Figures and Tables

**Table 1:** Chemical compositions of cement.

Chemical Composition	SiO_2_	Al_2_O_3_	Fe_2_O_3_	CaO	MgO	SO_3_	R_2_O	Others
Mass fraction (%)	20.03	4.69	4.74	63.42	1.36	2.85	1.45	1.46

**Table 2:** Mineralogical compositions of cement.

Mineral Composition	C_3_S	C_2_S	C_3_A	C_4_AF	Others
Mass fraction (%)	53.12	22.80	7.41	13.00	3.67

**Table 3:** Physical properties of cement.

Fineness (m^2^/kg)	Density (kg/m^3^)	Setting Time (min)		Flexural Strength (MPa)		Compressive Strength (MPa)	
		Initial	Final	3d	28d	3d	28d
335	3120	204	267	5.5	9.8	27.3	54.2

**Table 4:** Mix proportions of cement with nano-TiO_2_.

Code	Cement (g)	Water-Reducing Agent Content (%)	Nano-TiO_2_ Content (%)	Air-Entraining Agent Content (%)	Water-Cement Ratio
C0-0-0.4	100	2	0	0	0.40
C3-0-0.4	100	2	3	0	0.40
C6-0-0.4	100	2	6	0	0.40
C9-0-0.4	100	2	9	0	0.40
C12-0-0.4	100	2	12	0	0.40
C6-0.05-0.4	100	2	6	0.05	0.40
C6-0.1-0.4	100	2	6	0.10	0.40
C6-0.15-0.4	100	2	6	0.15	0.40
C6-0.2-0.4	100	2	6	0.20	0.40
C6-0-0.35	100	2	6	0	0.35
C6-0-0.45	100	2	6	0	0.45

Note: Taking C6-0.1-0.4 as an example, 6 indicates the nano-TiO_2_ content is 6% by mass of cement, 0.1 indicates the air-entraining agent content is 0.1% by mass of cement, and 0.4 indicates the water-cement ratio is 0.4.

**Table 5:** Apparent kinetic parameters for photocatalytic NO removal by nano-TiO_2_-modified cement pastes.

Sample Code	Apparent Pseudo-First-Order Model			Apparent Pseudo-Second-Order Model			Better-Fitting Model
	k_1_ (min^−1^)	R^2^	RMSE (ppb)	k_2_ (ppb^−1^ × min^−1^)	R^2^	RMSE (ppb)	
C3-0-0.4	0.4344	0.9928	0.63	0.00966	0.9886	0.80	Pseudo-first-order
C6-0-0.4	0.2849	0.9978	0.37	0.00412	0.9967	0.45	Pseudo-first-order
C9-0-0.4	0.4696	0.9940	0.75	0.00871	0.9902	0.95	Pseudo-first-order
C12-0-0.4	0.6889	0.9995	0.41	0.00928	0.9975	0.96	Pseudo-first-order

Note: Kinetic fitting was performed using the first 5 min after UV irradiation. The control sample C0-0-0.4 was excluded because it contained no nano-TiO_2_ photocatalyst.

**Table 6:** Calculated weight loss fractions of four compositions.

Code	C–S–H Gel	Ca(OH)_2_	CaCO_3_	Ca(NO_3_)_2_
C0-0-0.4	5.88	2.59	4.20	0.51
C6-0-0.4	6.33	3.17	4.92	0.59
C12-0-0.4	6.17	2.91	4.64	0.56

## Data Availability

The original contributions presented in this study are included in the article. Further inquiries can be directed to the corresponding author.
